# External validation reveals setting-dependent performance of TyG-related indices for HbA1c-defined prediabetes

**DOI:** 10.3389/fendo.2026.1961688

**Published:** 2026-10-08

**Authors:** Fen Li, Yingjing Xie, Yu Chen, Zexiu Wang, Chunmei Rao, Jinliu Cheng, Tianwu Zhang, Jin Yuan

**Affiliations:** Laboratory Department, Pu’er Hospital of Traditional Chinese Medicine, Pu’er, Yunnan, China

**Keywords:** external validation, NHANES, prediabetes, triglyceride–glucose index, TyG-WHtR

## Abstract

**Background and objective:**

Prediabetes defined by glycated haemoglobin (HbA1c) can occur despite fasting plasma glucose (FPG) below 100 mg/dL, leaving a discordant glycaemic phenotype that FPG-based pathways may miss. We investigated whether triglyceride–glucose (TyG)-related indices identify this phenotype and whether their comparative performance is maintained across populations.

**Methods:**

We analysed fasting-subsample data from seven US National Health and Nutrition Examination Survey (NHANES 2005–2018) cycles and an independent health-examination cohort from Pu’er Hospital of Traditional Chinese Medicine (2020–2026). The primary index was TyG–waist-to-height ratio (TyG–WHtR). Survey-weighted and local regression models quantified adjusted associations. Candidate prediction models were benchmarked against related metabolic indices and a model retaining FPG, triglycerides and WHtR as separate terms. Repeated cross-validation was performed in NHANES, after which all fitted models, transformations and thresholds were frozen for external validation.

**Results and conclusion:**

Among 6,930 eligible NHANES participants, 1,206 had HbA1c-defined prediabetes, corresponding to a weighted prevalence of 12.7% [95% confidence interval (CI), 11.7%–13.7%]. The local cohort included 10,791 participants and 1,139 outcomes. Each cohort-specific standard deviation increase in TyG–WHtR was associated with higher outcome prevalence in NHANES (prevalence ratio, 1.239; 95% CI, 1.158–1.326) and locally (1.482; 1.407–1.561). The raw-component model showed repeated internal validation (area under the receiver operating characteristic curve, 0.746), whereas the metabolic score for insulin resistance led to frozen external validation (0.710; 0.693–0.727). TyG–WHtR was reproducibly associated with the outcome but was not the strongest discriminator in either setting. The gains in discrimination were modest, and this cross-sectional validation does not establish clinical utility.

## Introduction

Prediabetes encompasses glycaemic states above normal but below diagnostic thresholds for diabetes. Contemporary criteria use fasting plasma glucose (FPG), 2-h glucose after an oral glucose challenge or glycated haemoglobin (HbA1c), and these tests capture related but non-identical biology (1–3). Chinese guidance likewise recognises distinct glycaemic criteria and recommends risk-based testing with appropriately standardised assays (4, 5). Hundreds of millions of adults worldwide meet an impaired fasting glucose or impaired glucose tolerance definition (6). Prediabetes is associated with diabetes progression, cardiovascular disease and mortality, although absolute risks vary by definition and population (7–9). Lifestyle intervention can reduce progression in selected high-risk adults (10).

Test discordance creates a distinct practical problem. HbA1c reflects longer-term glycaemic exposure and is associated with subsequent diabetes and cardiovascular outcomes among adults without diagnosed diabetes (11). However, FPG, 2-h glucose and HbA1c make different contributions to prediabetes prevalence (12). HbA1c can also differ at comparable glucose concentrations across population groups and can be altered by erythrocyte turnover or assay conditions (13, 14). Adults with HbA1c of 5.7 to <6.5% but FPG below 100 mg/dL therefore represent a reproducible phenotype, but not all possible forms of prediabetes. Routinely available metabolic measures might help prioritise HbA1c testing, provided that their incremental value and transportability are established.

The triglyceride–glucose (TyG) index combines fasting triglycerides and FPG as a non-insulin marker of insulin resistance (15). TyG has been compared with clamp-based insulin-sensitivity measures in Mexican and Brazilian populations (16, 17). Multiplication by body mass index (BMI), waist circumference or waist-to-height ratio (WHtR) adds an adiposity component, and TyG–BMI has also shown agreement with insulin-resistance phenotypes (18). Prospective cohorts collectively associate higher TyG with incident type 2 diabetes, although effect sizes and thresholds vary (19). WHtR is appealing because it expresses central adiposity relative to body size and has performed at least comparably with BMI in several systematic reviews and meta-analyses (20–23).

TyG–WHtR has shown strong cross-sectional discrimination for prediabetes in normal-weight Qatari adults (24). Chinese CHARLS data also linked TyG–WHtR with diabetes, whereas a Chinese health-examination study evaluated TyG–BMI in relation to recovery from FPG-defined prediabetes (25, 26). These outcomes differ from HbA1c-defined prediabetes with normal FPG. Prospective Japanese and fatty liver studies have associated TyG–WHtR with incident diabetes (27, 28). Nevertheless, a composite index should be compared with its constituent measurements (29). Metabolic Score for Insulin Resistance (METS-IR) provides a further comparator with evidence for insulin sensitivity and incident diabetes (30, 31). Prediabetes models may also require recalibration when transferred between health systems (32).

We, therefore, evaluated TyG–WHtR among adults with FPG below 100 mg/dL in two independent cohorts. We estimated adjusted associations and compared TyG–WHtR with related indices and raw components. We then quantified internal model performance in the National Health and Nutrition Examination Survey (NHANES) and externally validated frozen models in the local cohort. We expected a positive association in both cohorts, but treated comparative ranking as an empirical question.

The analysis separates association, incremental discrimination and performance after transfer between settings. Survey-weighted estimation, comparisons with raw components and frozen external validation address these questions within the limits of cross-sectional data.

## Methods

### Study design and reporting

This cross-sectional study combined association analysis, model comparison, internal validation and independent external validation. Analyses added during peer review are identified as supplementary or *post hoc*. Reporting followed Transparent Reporting of a Multivariable Prediction Model for Individual Prognosis or Diagnosis (TRIPOD) principles where applicable, with Prediction Model Risk of Bias Assessment Tool (PROBAST) domains informing the assessment of bias and applicability (33, 34).

### NHANES derivation sample

We combined seven continuous NHANES cycles from 2005–2006 through 2017–2018. NHANES uses a stratified, multistage probability design to represent the non-institutionalised US population (35). We used the fasting subsample to support comparisons involving glucose and triglycerides. Following National Center for Health Statistics (NCHS) guidance for equally long continuous cycles, 2-year fasting weights were divided by seven (36). These weights estimate population averages over the combined period. Masked strata and primary sampling units were retained, and survey design objects preceded primary subpopulation restriction. We checked invariance to retaining unscaled 2-year weights and estimated cycle-specific prevalence and associations (**Supplementary Table 10**). A constant weight rescaling does not address changes in population composition or outcome prevalence over time.

NHANES August 2021–August 2023 was analysed separately as a temporal sensitivity sample. It was not pooled with the primary sample because post-pandemic sampling and selected measurement procedures differed from earlier continuous cycles.

### Local external validation sample

The local dataset contained 15,498 routine health-examination records from Pu’er Hospital of Traditional Chinese Medicine, Yunnan, China, collected during 2020–2026. The primary sample applied the same age, diabetes, pregnancy, FPG and HbA1c criteria as NHANES. This was an independent geographical and healthcare-setting validation. It was not a representative validation sample for China. NHANES transformations, centering, scaling, coefficients and operating thresholds were applied unchanged. Local outcomes were not used to reselect predictors, refit coefficients or optimise thresholds. Direct identifiers and exact examination dates were absent from processed files.

Routine health-examination records were treated as one observation per included participant in the processed analytic file. Eligibility was applied independently in each cohort before the fixed common samples for model comparison were constructed. This separation preserved the full eligible samples for association analysis while ensuring that every candidate model was evaluated in exactly the same participants.

All local blood samples, including those used for HbA1c measurement, were collected after an overnight fast and analysed under the hospital’s standard operating procedures, which did not change during the study period. HbA1c was measured enzymatically on a Mindray BS-2800M analyser using reagents supplied by Shenzhen Mindray Bio-Medical Electronics Co., Ltd. (Shenzhen, Guangdong, China). FPG was measured using the hexokinase method, triglycerides using a colourimetric method, high-density lipoprotein cholesterol using an enzymatic colourimetric method and creatinine using an enzymatic method on a cobas c 702 analyser using reagents supplied by Roche Diagnostics (Indianapolis, IN, USA). Fasting insulin was measured via electrochemiluminescence on a cobas e 602 analyser using reagents supplied by Roche Diagnostics GmbH. The laboratory participated in the external quality-assessment programme organised by Yunnan Province. Blood pressure was measured using a Yuwell YE670A monitor (Danyang, Jiangsu, China). Height and weight were measured using a CKN/TCS-160D-W/H instrument manufactured by Shenzhen Shuangjia Electronics Co., Ltd. (Shenzhen, Guangdong, China).

### Eligibility and outcome definition

Participants were required to be at least 20 years old. We excluded pregnancy, self-reported physician-diagnosed diabetes and laboratory-defined diabetes, defined as FPG of at least 126 mg/dL or HbA1c of at least 6.5%. Planned range checks removed non-positive or implausible values. The primary population additionally required FPG below 100 mg/dL and a measured HbA1c. The binary outcome was HbA1c from 5.7% to <6.5%, with HbA1c below 5.7% as the control state (1, 2). We used “HbA1c-defined prediabetes despite normal FPG” because oral glucose tolerance was not measured consistently.

The primary sensitivity analysis increased the FPG ceiling to 110 mg/dL, corresponding to the historical World Health Organization definition of impaired fasting glycaemia (3). A stricter analysis used FPG below 95 mg/dL.

### Metabolic indices

Glucose and lipid concentrations were expressed in mg/dL unless stated otherwise. We calculated TyG as ln(triglycerides × FPG/2). Derived indices were TyG–BMI = TyG × BMI, TyG–WC = TyG × waist circumference in centimetres and TyG–WHtR = TyG × (waist circumference/height). METS-IR was ln(2 × FPG + triglycerides) × BMI/ln(high-density lipoprotein cholesterol). The cardiometabolic index was (triglycerides/high-density lipoprotein cholesterol in mmol/L) × WHtR. We also calculated triglycerides/high-density lipoprotein cholesterol.

TyG–WHtR was the planned primary index. Association comparisons included TyG, TyG–BMI, TyG–WC, METS-IR, cardiometabolic index, triglycerides/high-density lipoprotein cholesterol, FPG, triglycerides, BMI and WHtR. Continuous association estimates were scaled per cohort-specific standard deviation. All prediction transformations and scaling values were estimated in NHANES only.

### Candidate models and covariates

The prediction reference model contained age, sex, systolic blood pressure and smoking. Candidate models separately added FPG, triglycerides, TyG, TyG–BMI, TyG–WC, TyG–WHtR, METS-IR or cardiometabolic index. The raw-component comparator added FPG, triglycerides and WHtR separately. **Supplementary Table 8** lists candidate-specific data availability, actual common-sample counts and events for every model. BMI-only and WHtR-only additions were evaluated as secondary comparisons requested during peer review, using the same common samples and validation folds (**Supplementary Table 16**).

Association Model 1 adjusted for age, sex and NHANES cycle or local enrolment year. Model 2 additionally included education, income, smoking, alcohol use and vigorous activity in NHANES or physical activity locally. Model 3 additionally included systolic blood pressure, hypertension, lipid-lowering medication and estimated glomerular filtration rate (eGFR). Directly recorded family history of diabetes was included in local Model 3. It was omitted from the cross-cycle NHANES model because no valid and consistently collected yes/no item was available across 2005–2018. eGFR was derived using the 2021 Chronic Kidney Disease Epidemiology Collaboration (CKD-EPI) creatinine equation without race (37).

### Association analysis

NHANES associations were estimated using survey-weighted modified Poisson’s regression with a log link and design-based standard errors. Local associations used modified Poisson’s regression with HC0 sandwich standard errors (38). Restricted cubic splines used four knots at the survey-weighted 5th, 35th, 65th and 95th percentiles of TyG–WHtR. A Wald’s test assessed the joint non-linear terms. Logistic odds ratios were estimated only as a sensitivity analysis.

### Missing data

In the primary association analysis, covariates were multiply imputed by chained equations using 50 imputations and 20 iterations (39). Continuous covariates used predictive mean matching, binary covariates logistic regression and multicategory covariates polytomous regression. Outcomes and index components were not imputed in this analysis. Survey-weighted or sandwich estimates were combined using Rubin’s rules with finite-degree-of-freedom adjustment. We examined chain traces, logged events and observed-versus-imputed distributions. Complete-case estimates were secondary. During peer review, we additionally compared participants with observed and missing index components and assessed selection weighting (**Supplementary Tables 9**, **12**, **13**). Separate outcome-imputation sensitivities are described below.

### Internal validation and model comparison

Candidate models were compared on a fixed NHANES common sample using 10-fold cross-validation repeated five times. The entire primary sampling units were assigned to folds, and all models used the same splits. **Table 1** reports mean performance across repeats. Averaged out-of-fold predictions were used for curves, paired area under the receiver operating characteristic (ROC) curve (AUC) contrasts and threshold selection. ROC AUC, precision–recall AUC, Brier score and calibration summaries capture complementary aspects of performance (40, 41). **Supplementary Table 8** also reports training and held-out counts and events for each fold.

**Table 1:** Repeated internal validation in the fixed NHANES common sample.

Model	ROC AUC	PR AUC	Brier score	Calibration intercept	Calibration slope	ΔAUC vs. reference (95% CI)	ΔAUC vs. FPG + TG + WHtR (95% CI)
Reference	0.727	0.268	0.102	−0.035	0.98	Reference	−0.0192 (−0.0284 to −0.0100)
FPG + TG + WHtR	0.746	0.289	0.1	−0.045	0.974	0.0192 (0.0093 to 0.0291)	Reference
METS-IR	0.741	0.288	0.1	−0.037	0.978	0.0140 (0.0059 to 0.0227)	−0.0053 (−0.0118 to 0.0011)
TyG–BMI	0.74	0.285	0.1	−0.039	0.978	0.0132 (0.0054 to 0.0214)	−0.0061 (−0.0117 to −0.0002)
TyG–WHtR	0.739	0.283	0.1	−0.039	0.978	0.0119 (0.0044 to 0.0202)	−0.0073 (−0.0131 to −0.0016)

All candidates include the reference covariates of age, sex, systolic blood pressure and smoking, plus the variables named in the row. The same 6,455 participants and 1,116 outcomes are used throughout. Metrics are means across five repeats of 10-fold PSU-grouped cross-validation. AUC differences use averaged out-of-fold predictions and 1,000 PSU cluster-bootstrap replicates. The calibration intercept is estimated jointly with the slope.

NHANES, National Health and Nutrition Examination Survey; ROC, receiver operating characteristic; AUC, area under the curve; FPG, fasting plasma glucose; TG, triglyceride; WHtR, waist-to-height ratio; TyG, triglyceride–glucose; BMI, body mass index; PSU, primary sampling unit.

A 1,000-replicate primary-sampling-unit cluster bootstrap provided 95% confidence intervals (CIs) for paired internal ROC AUC differences. The study was not prospectively sized; external precision was characterised by confidence intervals rather than a universal event-count target (42). Precision–recall curves were included because the outcome was less common than the control state (43). Decision curves evaluated the net benefit of ordering an HbA1c test across thresholds ranging from 5% to 25% (44).

For each model, a survey-weighted Youden’s threshold was selected once from averaged repeated out-of-fold NHANES predictions. The threshold was frozen before external application. Threshold metrics describe derivation-dependent transportability and are not recommended clinical cut-offs.

The added NHANES Inverse Probability Weighting (IPW) analysis modelled inclusion in the common prediction sample among all eligible participants with observed HbA1c. Selection models used age, sex, cycle, FPG and race or ethnicity and were estimated within training primary sampling units. We compared original predictions reweighted at evaluation with models reweighted during both training and evaluation. These analyses address measured predictors of complete-record selection and do not recover discrimination in people whose predictors remain unobserved without assumptions.

### External validation

Full candidate models were fitted in the NHANES common sample using survey weights. Frozen predictions were then generated for a fixed local common sample. We reported ROC AUC with DeLong 95% CIs, precision–recall AUC, Brier score, calibration intercept and calibration slope (45). Frozen-threshold summaries included sensitivity, specificity, positive predictive value and negative predictive value. Paired DeLong tests compared external ROC AUCs. Intercept-only recalibration was secondary and was separated from primary frozen validation.

### Subgroup, sensitivity and convergent analyses

Subgroups were sex, age younger than 50 versus at least 50 years, BMI, hypertension, lipid-lowering therapy and NHANES race or ethnicity. The original BMI groups were <25, 25 to <30 and ≥30 kg/m^2^ in both cohorts. A post-hoc local sensitivity analysis used Chinese thresholds of <24, 24 to <28, and ≥28 kg/m^2^ (4). Subgroup models were adjusted for age, sex and cycle or enrolment year, with age omitted for age strata and sex omitted for sex strata. The original interaction family used the Benjamini–Hochberg false discovery rate adjustment (46); the additional BMI analysis is reported separately. Unadjusted prevalence and population composition were summarised across cohort-wide TyG–WHtR quartiles, fixed before sex or age stratification. Sensitivity analyses also used alternative FPG ceilings, treatment and kidney function restrictions, logistic models and local calendar periods.

Where fasting insulin was available, homeostasis model assessment of insulin resistance (HOMA-IR) was calculated using fasting glucose and insulin levels (47). We estimated correlations with log HOMA-IR and adjusted geometric-mean ratios across TyG–WHtR. Because HOMA-IR and TyG share fasting glucose, this analysis was designated convergent rather than mechanistic evidence.

Local HbA1c-availability weights were estimated among adults meeting eligibility criteria ascertainable before HbA1c measurement, using age, sex, enrolment year and FPG (48). We reported weight ranges, effective sample size and covariate balance before and after weighting, relative to this target population. The original Standardized Mean Difference (SMD) of 0.047 compared participants with and without HbA1c before weighting in a broader FPG < 126 mg/dL population; it was not a post-IPW balance statistic. Frozen local model predictions were also evaluated with availability weights and with weights truncated at the 1st and 99th percentiles (**Supplementary Tables 12**, **13**).

For outcome-imputation sensitivity, we retained adults with FPG < 100 mg/dL and measured TyG–WHtR components, including those without HbA1c. HbA1c and missing covariates were imputed in 50 datasets over 20 iterations. HbA1c was imputed using predictive mean matching with the index, covariates and FPG as predictors, plus log survey weight in NHANES. Index components were not imputed. We shifted only missing HbA1c values from −0.5 to +0.5 percentage points in 0.1-point steps, reclassified the outcome and excluded HbA1c ≥ 6.5% within each dataset. Models used the original cohort-specific index scaling. Rubin-pooled associations describe a Missing at Random (MAR) analysis and a pattern-mixture stress test, not evidence that the assumed missingness mechanisms are correct (**Supplementary Table 14**).

All reported tests were two-sided. Exact P-values were retained unless they were smaller than the reporting precision of the corresponding table or figure. Analyses of subgroup interaction, alternative glucose thresholds, treatment exclusion, kidney function restriction, calendar periods and HOMA-IR convergence were interpreted as supportive or exploratory rather than as independent confirmation of the primary prediction claim.

### Software and reproducibility

Analyses used R version 4.5.1 with survey, sandwich, mice, pROC, PRROC, data.table, ggplot2 and related packages. The original workflow used seed 2026; added outcome imputations used seeds 260908 and 260909. The reproducibility package contains scripts, the download manifest, saved model objects, source data, tables and session information.

## Results

### Two cohorts captured the same discordant glycaemic phenotype

The primary NHANES sample combined seven survey cycles from 2005–2006 through 2017–2018. Among 70,190 examination records, 20,078 had positive fasting weights, and 6,930 met the eligibility criteria (**Figure 1a**). Of these, 1,206 had HbA1c-defined prediabetes despite FPG below 100 mg/dL. Survey-weighted prevalence was 12.7% (95% CI, 11.7–13.7%). TyG–WHtR was complete in 6,651 participants, including 1,152 outcomes. Thus, 279 lacked TyG–WHtR components; the common prediction sample excluded 475 participants due to missingness of any required predictor or covariate. Their characteristics are reported in **Supplementary Table 9**.

**Figure 1:**
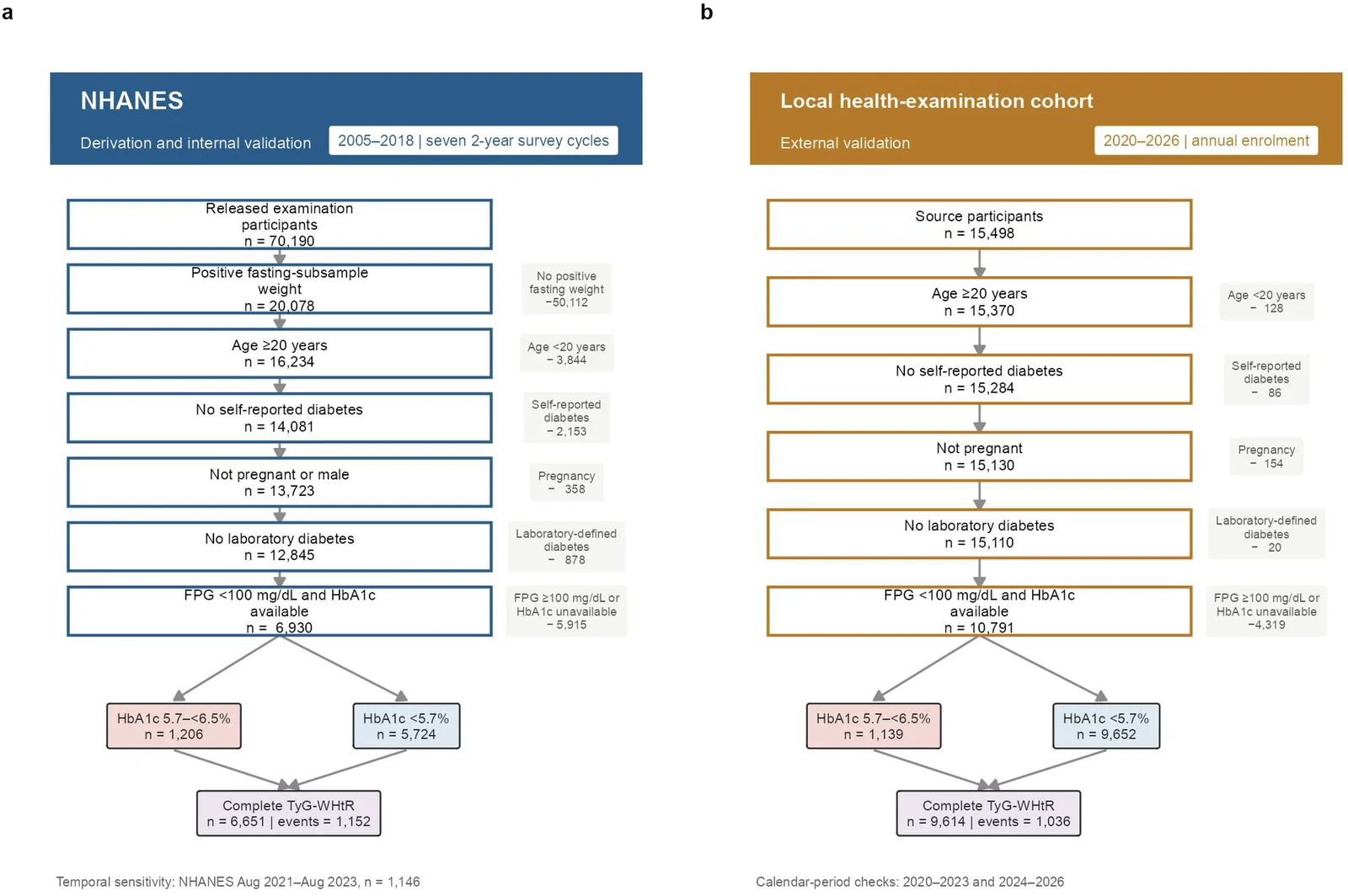
Construction and temporal coverage of the derivation and external-validation cohorts. **(a)** NHANES derivation and internal validation sample across seven 2-year survey cycles from 2005 through 2018. **(b)** Local health-examination external-validation cohort enrolled from 2020 through 2026. Boxes show unweighted participant counts after each criterion, while side labels show exclusions at that step. The outcome split is HbA1c 5.7 to <6.5% versus <5.7% among adults with FPG < 100 mg/dL. Complete TyG–WHtR samples supported the primary association analysis. NHANES August 2021–August 2023 and the local 2020–2023 and 2024–2026 periods were analysed as temporal sensitivity samples. Source data are provided with the study files. NHANES, National Health and Nutrition Examination Survey; FPG, fasting plasma glucose; TyG, triglyceride–glucose; WHtR, waist-to-height ratio.

The health-examination cohort from Pu’er Hospital of Traditional Chinese Medicine enrolled participants from 2020 through 2026. Among 15,498 source records, 10,791 met the same primary criteria, and 1,139 (10.6%) had the outcome (**Figure 1b**). TyG–WHtR was complete for 9,614 participants, including 1,036 outcomes. NHANES 2021–2023 was reserved for temporal sensitivity analysis, while the Pu’er cohort was also divided into 2020–2023 and 2024–2026 calendar periods.

Participants with outcomes were older and had less favourable metabolic profiles in both cohorts (**Table 2**). In NHANES, their weighted mean age was 53.0 years, compared with 40.6 years among controls. Corresponding BMI values were 28.6 and 26.9 kg/m^2^, while FPG values were 93.3 and 91.6 mg/dL. Triglyceride values were 117.0 and 105.5 mg/dL, and TyG–WHtR values were 5.0 and 4.6. The local cohort showed similar gradients, with mean ages of 56.8 and 48.7 years and TyG–WHtR values of 4.39 and 4.10.

**Table 2:** Baseline characteristics by HbA1c outcome status.

Cohort	Characteristic	Overall	HbA1c <5.7%	HbA1c 5.7 to <6.5%
NHANES 2005–2018	Age, years	42.2 (95% CI 41.6–42.7)	40.6 (95% CI 40.0–41.2)	53.0 (95% CI 51.8–54.3)
	Female, %	59.0% (95% CI 57.7–60.4)	58.6% (95% CI 57.1–60.0)	62.0% (95% CI 57.9–66.1)
	Body mass index, kg/m^2^	27.1 (95% CI 26.9–27.3)	26.9 (95% CI 26.6–27.1)	28.6 (95% CI 28.2–29.1)
	Waist circumference, cm	93.2 (95% CI 92.6–93.8)	92.6 (95% CI 92.0–93.2)	97.6 (95% CI 96.5–98.7)
	Fasting plasma glucose, mg/dL	91.8 (95% CI 91.6–92.0)	91.6 (95% CI 91.4–91.8)	93.3 (95% CI 92.8–93.8)
	HbA1c, %	5.3 (95% CI 5.3–5.3)	5.2 (95% CI 5.2–5.2)	5.8 (95% CI 5.8–5.8)
	Triglycerides, mg/dL	106.9 (95% CI 104.6–109.3)	105.5 (95% CI 103.0–108.0)	117.0 (95% CI 110.7–123.3)
	HDL cholesterol, mg/dL	57.4 (95% CI 56.9–57.9)	57.5 (95% CI 56.9–58.0)	56.9 (95% CI 55.5–58.2)
	Systolic blood pressure, mmHg	117.2 (95% CI 116.6–117.7)	116.2 (95% CI 115.7–116.8)	123.7 (95% CI 122.5–125.0)
	Current smoking, %	21.4% (95% CI 19.7–23.0)	20.7% (95% CI 18.9–22.5)	25.9% (95% CI 22.3–29.5)
	Hypertension, %	21.2% (95% CI 19.8–22.6)	18.8% (95% CI 17.3–20.3)	37.5% (95% CI 34.1–40.8)
	Lipid-lowering therapy, %	9.9% (95% CI 8.8–11.0)	7.9% (95% CI 6.8–8.9)	22.8% (95% CI 19.6–26.1)
	TyG–WHtR	4.6 (95% CI 4.6–4.7)	4.6 (95% CI 4.5–4.6)	5.0 (95% CI 4.9–5.0)
	METS-IR	38.7 (95% CI 38.3–39.0)	38.3 (95% CI 37.9–38.7)	41.3 (95% CI 40.4–42.1)
Local cohort	Age, years	49.6 (12.5)	48.7 (12.3)	56.8 (11.9)
	Female, %	51.10%	51.00%	51.90%
	Body mass index, kg/m^2^	24.1 (3.5)	23.9 (3.5)	25.3 (3.4)
	Waist circumference, cm	79.1 (10.5)	78.7 (10.4)	82.1 (10.5)
	Fasting plasma glucose, mg/dL	84.6 (9.4)	84.3 (9.5)	87.2 (8.5)
	HbA1c, %	5.2 (0.4)	5.2 (0.3)	5.9 (0.1)
	Triglycerides, mg/dL	129.5 (65.3)	126.4 (63.8)	154.9 (71.6)
	HDL cholesterol, mg/dL	50.7 (13.3)	51.3 (13.3)	46.3 (12.2)
	Systolic blood pressure, mmHg	120.0 (14.9)	119.6 (14.9)	123.4 (15.0)
	Current smoking, %	23.70%	23.70%	23.40%
	Hypertension, %	7.90%	7.40%	11.60%
	Lipid-lowering therapy, %	4.20%	4.10%	4.80%
	TyG–WHtR	4.1 (0.6)	4.1 (0.6)	4.4 (0.6)
	METS-IR	35.3 (6.5)	34.9 (6.4)	38.7 (6.7)

NHANES values are survey-weighted and shown with 95% CIs; local values are unweighted summaries.

HbA1c, glycated haemoglobin; NHANES, National Health and Nutrition Examination Survey; HDL, high-density lipoprotein; TyG, triglyceride–glucose; WHtR, waist-to-height ratio.

### TyG–WHtR was associated with outcome prevalence in both cohorts

After multiple imputation and full adjustment, each standard deviation increase in TyG–WHtR was associated with 23.9% higher outcome prevalence in NHANES. The Prevalence Ratio (PR) was 1.239 (95% CI, 1.158–1.326; two-sided P = 8.45×10^-9^) (**Table 3**; **Figure 2a**) . Local outcome prevalence was 48.2% higher per standard deviation (PR, 1.482; 95% CI, 1.407–1.561; two-sided P = 1.16×10^-49^). Estimates changed little across the three adjustment sets. Complete-case estimates were directionally consistent in NHANES (PR, 1.250; 95% CI, 1.154–1.355) and locally (PR, 1.491; 95% CI, 1.396–1.593).

**Table 3:** Association of TyG–WHtR with HbA1c-defined prediabetes.

Cohort	Model	n	Outcomes	PR per SD (95% CI)	P-value	Imputations
NHANES 2005–2018	Model 1	6,651	1,152	1.298 (1.217–1.385)	<0.001	50
NHANES 2005–2018	Model 2	6,651	1,152	1.261 (1.181–1.347)	<0.001	50
NHANES 2005–2018	Model 3	6,651	1,152	1.239 (1.158–1.326)	<0.001	50
Local cohort	Model 1	9,614	1,036	1.480 (1.406–1.558)	<0.001	50
Local cohort	Model 2	9,614	1,036	1.481 (1.407–1.559)	<0.001	50
Local cohort	Model 3	9,614	1,036	1.482 (1.407–1.561)	<0.001	50

Model 1 adjusts for age, sex and NHANES cycle or local enrolment year. Model 2 additionally adjusts for education, income-to-poverty ratio in NHANES or income category locally, smoking, alcohol use and vigorous activity in NHANES or physical activity locally. Model 3 additionally adjusts for systolic blood pressure, hypertension, lipid-lowering medication and eGFR, with family history of diabetes included locally. PRs are per cohort-specific standard deviation increase. NHANES uses design-based standard errors; local estimates use HC0 sandwich standard errors. Primary estimates use multiple imputation of covariates only.

TyG, triglyceride–glucose; WHtR, waist-to-height ratio; HbA1c, glycated haemoglobin; NHANES, National Health and Nutrition Examination Survey.

**Figure 2:**
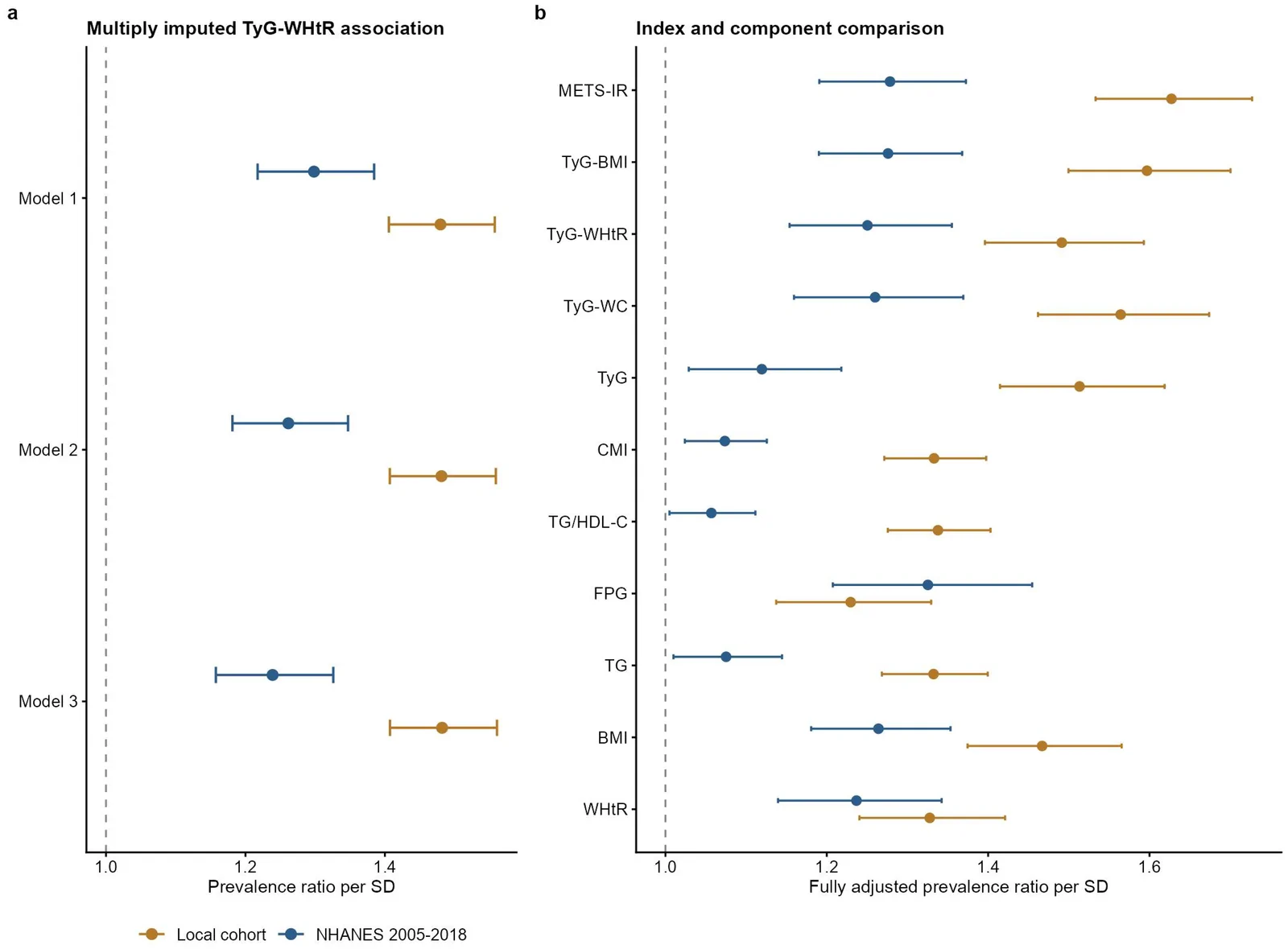
TyG–WHtR associations and comparison with related metabolic indices. **(a)** Multiply imputed TyG–WHtR prevalence ratios across adjustment Models 1–3 in NHANES and the local cohort. **(b)** Complete-case, fully adjusted prevalence ratios for selected indices and individual components. Points show prevalence ratios per cohort-specific standard deviation, and bars show 95% CIs. NHANES analyses used fasting-subsample weights and design-based standard errors. Panel b is exploratory, and effect size ranking does not establish discriminative superiority. Source data are provided with the study files. TyG, triglyceride–glucose; WHtR, waist-to-height ratio; NHANES, National Health and Nutrition Examination Survey.

The survey-weighted restricted cubic spline did not support departure from linearity across the central TyG–WHtR distribution (P for non-linearity = 0.706; **Figure 3**). In fully adjusted complete-case comparisons, FPG produced the largest PR in NHANES (1.325 per standard deviation; 95% CI, 1.207–1.455), whereas METS-IR produced the largest PR locally (1.627; 95% CI, 1.533–1.727). TyG–WHtR ranked below METS-IR and TyG–BMI in both cohorts (**Figure 2b**). These effect sizes describe associations and were not used to infer superiority in individual-level discrimination.

**Figure 3:**
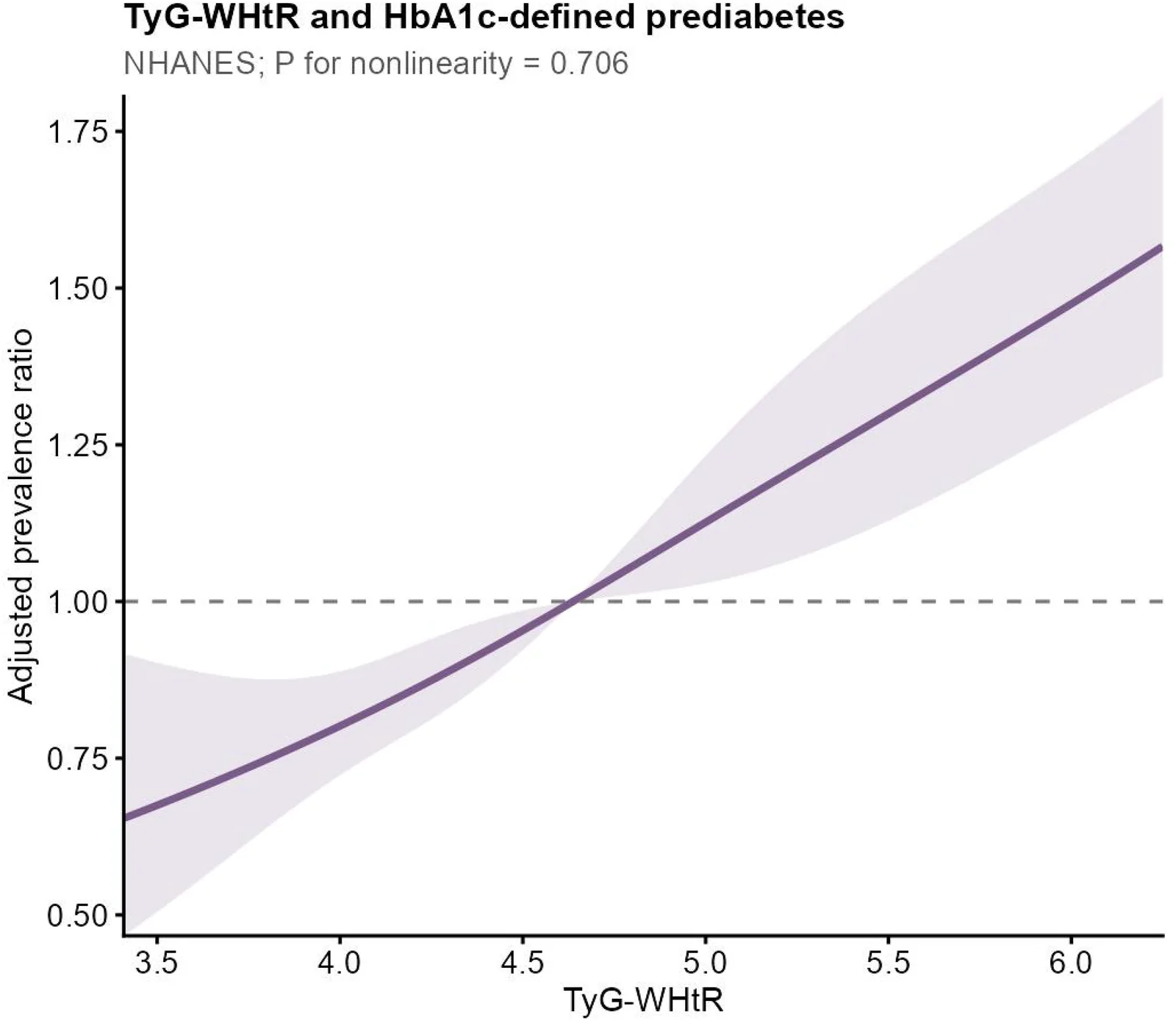
Association of TyG–WHtR with HbA1c-defined prediabetes in NHANES. The solid line shows the adjusted survey-weighted prevalence ratio from a restricted cubic spline, and shading denotes the 95% CI. The model used four knots at the survey-weighted 5th, 35th, 65th and 95th percentiles and adjusted for age, sex and survey cycle. The reference is the derivation-sample mean. The displayed range is restricted to the 5th–95th percentiles. Wald’s P-value for non-linearity is 0.706 (n = 6,651; 1,152 outcomes). Source data are provided with the study files. TyG, triglyceride–glucose; WHtR, waist-to-height ratio; HbA1c, glycated haemoglobin; NHANES, National Health and Nutrition Examination Survey.

### Raw components led internal discrimination

All candidate models used the same NHANES common sample of 6,455 participants, including 1,116 outcomes (**Supplementary Table 8**). The reference model produced a mean repeated cross-validated ROC AUC of 0.727, precision–recall AUC of 0.268, Brier score of 0.102 and calibration slope of 0.980 (**Table 1**). Adding FPG, triglycerides and WHtR separately gave the highest internal ROC AUC (0.746) and precision–recall AUC (0.289). METS-IR, TyG–BMI and TyG–WHtR yielded ROC AUCs of 0.741, 0.740 and 0.739, respectively. Secondary additions of BMI or WHtR alone each yielded an AUC of 0.740 (**Supplementary Table 16**).

No composite index exceeded the raw-component model internally. Relative to that model, the ROC AUC difference was −0.0053 (95% cluster-bootstrap CI, −0.0118 to 0.0011) for METS-IR. Differences were −0.0061 (−0.0117 to −0.0002) for TyG–BMI and −0.0073 (−0.0131 to −0.0016) for TyG–WHtR. The leading ROC and precision–recall curves remained close (**Figures 4a, b**). Decision curves showed only modest separation across thresholds from 5% to 25% (**Supplementary Figure 1**).

**Figure 4:**
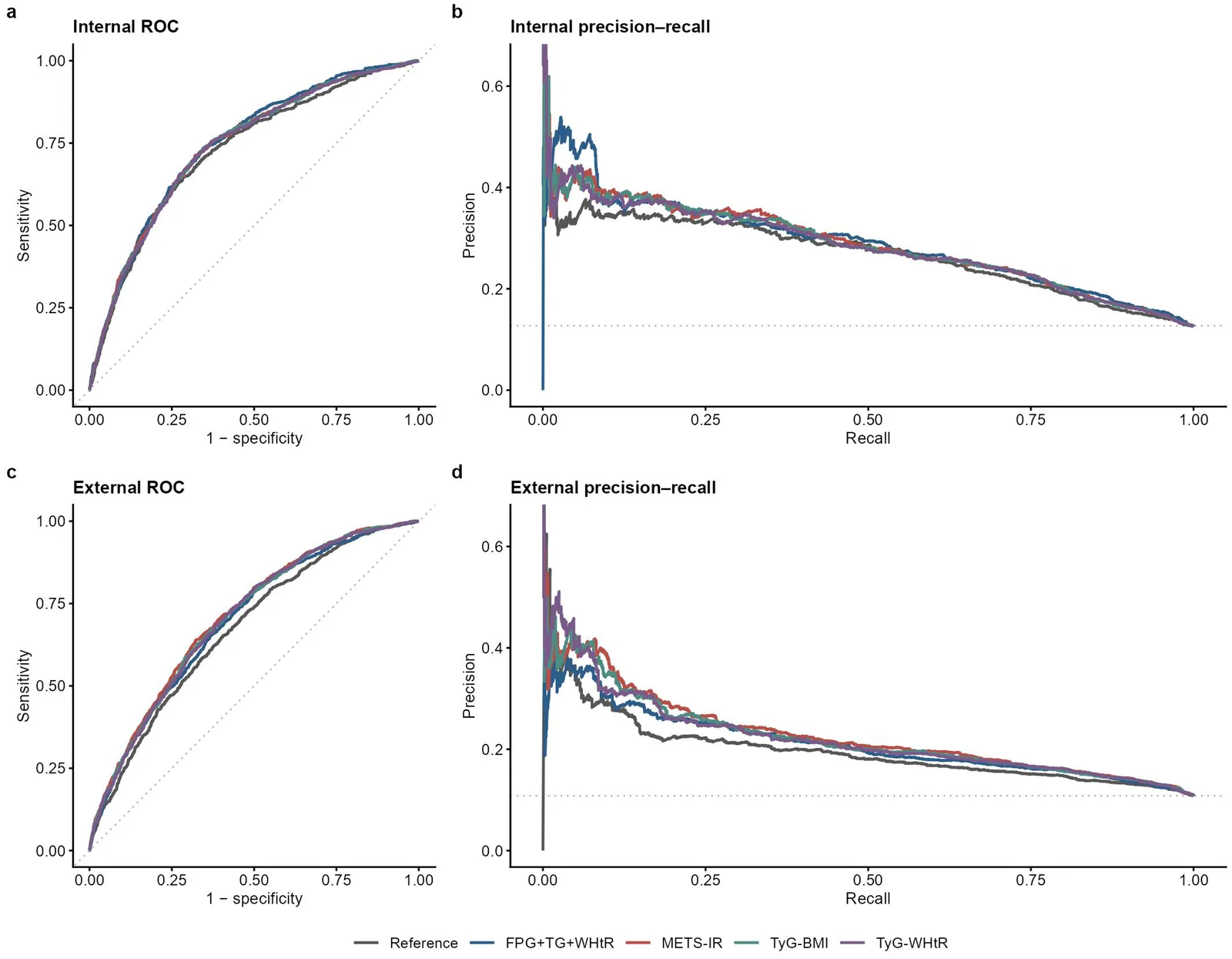
Internal and external discrimination of frozen candidate models. **(a, b)** Survey-weighted receiver operating characteristic and precision–recall curves from averaged out-of-fold NHANES predictions. Cross-validation used 10 folds repeated five times with entire primary sampling units assigned to folds (n = 6,455; 1,116 outcomes). **(c, d)** Receiver operating characteristic and precision–recall curves after frozen application to the local common sample (n = 8,574; 928 outcomes). Dotted horizontal lines in precision–recall panels indicate outcome prevalence. Internal uncertainty used 1,000 primary-sampling-unit cluster-bootstrap replicates, and external AUC comparisons used paired DeLong tests. Source data are provided with the study files. NHANES, National Health and Nutrition Examination Survey; AUC, area under the curve.

### Comparative ranking changed during external validation

All NHANES models were applied to the local common sample without local refitting, marker reselection, rescaling or threshold optimisation. The common external sample included 8,574 participants and 928 outcomes. METS-IR produced the highest external ROC AUC (0.710; 95% CI, 0.693–0.727) and precision–recall AUC (0.228) (**Table 4**; **Figures 4c, d**). TyG–BMI and TyG–WHtR yielded ROC AUC values of 0.704 (0.687–0.721) and 0.703 (0.686–0.720), respectively. The raw-component model yielded an ROC AUC of 0.695 (0.678–0.712) and a precision–recall AUC of 0.212.

**Table 4:** Frozen external validation in the local common sample.

Model	ROC AUC (95% CI)	PR AUC	Brier score	Calibration intercept	Calibration slope	ΔAUC vs. FPG + TG + WHtR (95% CI); P
Reference	0.672 (0.655–0.690)	0.196	0.097	−0.608	0.939	−0.0232 (−0.0348 to −0.0115); P<0.001
FPG + TG + WHtR	0.695 (0.678–0.712)	0.212	0.092	−0.302	0.83	Reference
METS-IR	0.710 (0.693–0.727)	0.228	0.093	−0.35	1.052	0.0148 (0.0048 to 0.0248); P = 0.004
TyG–BMI	0.704 (0.687–0.721)	0.223	0.093	−0.35	1.03	0.0084 (−0.0014 to 0.0182); P = 0.092
TyG–WHtR	0.703 (0.686–0.720)	0.222	0.092	−0.174	1.081	0.0078 (−0.0013 to 0.0169); P = 0.093

All candidates include age, sex, systolic blood pressure and smoking, plus the variables named in the row. All use the same 8,574 participants and 928 outcomes. NHANES coefficients, transformations and thresholds are applied without local fitting. AUC intervals and paired contrasts use DeLong methods. The calibration intercept is estimated jointly with the slope.

ROC, receiver operating characteristic; AUC, area under the curve; FPG, fasting plasma glucose; TG, triglyceride; WHtR, waist-to-height ratio; TyG, triglyceride–glucose; BMI, body mass index.

METS-IR exceeded the raw-component model externally by 0.0148 ROC AUC units (95% CI, 0.0048–0.0248; paired DeLong P = 0.0036). The corresponding differences for TyG–BMI (0.0084; 95% CI, −0.0014 to 0.0182; P = 0.092) and TyG–WHtR (0.0078; 95% CI, −0.0013 to 0.0169; P = 0.093) were not statistically distinguishable from zero. Thus, the internal leader was the raw-component model, whereas the external leader was METS-IR.

Calibration also differed between models (**Figure 5**). TyG–WHtR had an external calibration intercept of −0.174, a slope of 1.081 and a Brier score of 0.0924. The values were −0.302, 0.830 and 0.0921 for the raw-component model, and −0.350, 1.052 and 0.0933 for METS-IR. At the frozen NHANES TyG–WHtR threshold of 0.121, the sensitivity was 75.9%, specificity was 53.4%, positive predictive value was 16.5% and negative predictive value was 94.8%. This operating point characterises transportability and is not a clinical cut-off.

**Figure 5:**
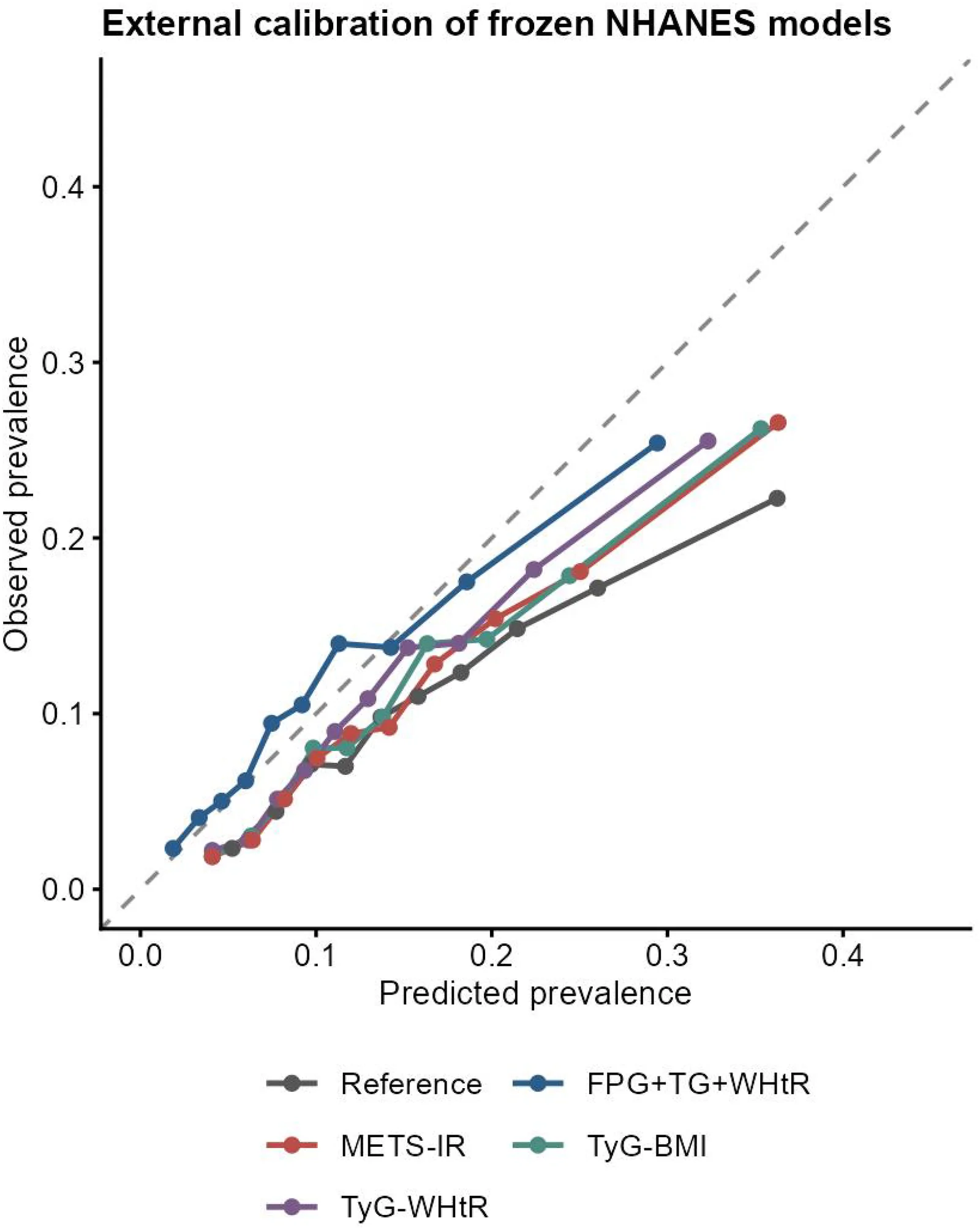
External calibration of frozen NHANES models. Points show observed outcome prevalence within local prediction groups, and lines connect groups for each model. The dashed diagonal denotes ideal calibration. The primary display contains frozen predictions without local refitting or rescaling (n = 8,574; 928 outcomes). Calibration intercepts and slopes are reported in **Table 4**. Source data are provided with the study files. NHANES, National Health and Nutrition Examination Survey.

### Sensitivity analyses supported association but not subgroup generalisation

Positive associations persisted with FPG ceilings of 110 and 95 mg/dL, treatment and kidney function restrictions, logistic models and local calendar-period analyses (**Supplementary Table 5**). The separate NHANES 2021–2023 estimate remained positive (PR, 1.510; 95% CI, 1.353–1.685; n = 1,146). Weight rescaling left pooled estimates unchanged; cycle-specific PRs ranged from 1.146 to 1.538, with no clear evidence of index-by-cycle interaction (P = 0.193; **Supplementary Table 10**).

Within the local pre-HbA1c target population, the maximum absolute SMD between observed participants and the target decreased from 0.039 before weighting to 0.006 after weighting. The corresponding values for NHANES common-sample selection were 0.015 and 0.004 (**Supplementary Table 12**; **Supplementary Figure 5**). Weighted refitting and evaluation using averaged predictions changed the internal TyG–WHtR AUC from 0.7387 to 0.7395. The local HbA1c weighting adjustment shifted the frozen AUC from 0.7031 to 0.7029 but left the leading model rankings unchanged (**Supplementary Table 13**). With missing HbA1c imputed under MAR, the fully adjusted PRs were 1.240 (95% CI, 1.159–1.328) in NHANES and 1.485 (1.409–1.565) locally. Positive associations persisted throughout the specified HbA1c-shift grid (**Supplementary Table 14**; **Supplementary Figure 8**).

NHANES interactions survived false discovery rate control for sex, age, hypertension and lipid-lowering therapy, but none survived locally. Associations were stronger among women and adults younger than 50 years in NHANES. BMI interaction tests did not support heterogeneity in NHANES (P = 0.096, FDR P = 0.116) or locally (P = 0.498, FDR P = 0.528). Local Chinese BMI thresholds yielded the same interpretation (interaction P = 0.435; **Supplementary Table 11**). Quartile prevalence and composition are shown in **Supplementary Table 15** and **Supplementary Figures 6** and **7**. These descriptive and interaction findings are hypothesis-generating and require confirmation in independent Chinese cohorts or prospective studies.

TyG–WHtR correlated with log-transformed HOMA-IR in NHANES (survey-weighted Pearson’s r = 0.591; n = 3,961) and locally (r = 0.360; n = 6,426). Each standard deviation increase in TyG–WHtR was associated with an adjusted HOMA-IR geometric-mean ratio of 1.551 (95% CI, 1.513–1.589) in NHANES and 1.205 (1.191–1.219) locally. METS-IR and TyG–BMI showed slightly stronger correlations in both cohorts (**Supplementary Figure 4**). Because these measures share fasting metabolic inputs, the findings provide convergent evidence rather than mechanistic validation.

## Discussion

Across two independent adult cohorts restricted to FPG below 100 mg/dL, higher TyG–WHtR was associated with HbA1c-defined prediabetes after multivariable adjustment. The association was approximately linear in NHANES and persisted across glucose thresholds, calendar periods, HbA1c-availability weighting and a separate NHANES 2021–2023 sample. Nevertheless, the comparative analyses rejected a universal TyG–WHtR advantage. Raw FPG, triglycerides and WHtR led internal discrimination, while METS-IR led external discrimination and was the only composite index that clearly exceeded the raw-component model locally.

Association and discrimination answer different questions. A prevalence ratio does not establish that an index adds the most useful individual-level information. TyG–WHtR imposes a fixed relationship between glucose, triglycerides and adiposity, whereas the raw-component model estimates their contributions separately. Restricting FPG also changes the glucose range and the glycaemic phenotype being classified. The internal advantage of separate components is compatible with these constraints, but does not identify information loss or outcome selection as its sole cause.

The external ranking shows that performance depends on setting. The US survey and Chinese health-examination cohort differ in age, adiposity, population composition, calendar period and measurement context. METS-IR may capture information that transfers differently across these case mixes (30, 31), but this study cannot isolate an ethnic or biological explanation for the ranking change. Its higher external ROC AUC also coexisted with a slightly higher Brier score than the raw-component model. The AUC gains were modest and must be considered alongside calibration and the consequences of missed outcomes.

Earlier studies evaluated normal-weight Qatari adults, Japanese cohorts and broader Chinese populations using different endpoints and comparator sets (24–28). Our FPG-restricted, HbA1c-defined outcome is narrower. Chinese studies have also distinguished prediabetes subgroups with different metabolic characteristics and subsequent outcomes (49). Association with this selected phenotype, therefore, cannot be assumed to generalise to all forms of prediabetes or to future diabetes.

The HOMA-IR findings also require cautious interpretation. TyG was developed and validated as a practical substitute for insulin resistance (15–17), while TyG–BMI and METS-IR add anthropometric or lipid information (18, 30). Nevertheless, HOMA-IR, TyG and METS-IR all use fasting glucose as a core component, and several indices also overlap in their use of triglycerides or BMI. Correlation, therefore, partly reflects mathematical coupling. The current outcome is HbA1c-defined glycaemic status rather than insulin resistance. The most defensible interpretation is that these indices summarise overlapping metabolic and adiposity information relevant to dysglycaemia.

External calibration showed why discrimination alone is insufficient. At the original frozen TyG–WHtR model threshold, retrospective triage would select 4,265 of 8,574 adults for HbA1c testing, detecting 704 of 928 outcomes and missing 224. This corresponds to approximately 497 tests and 26 missed outcomes per 1,000 adults (**Supplementary Table 17**). High negative predictive value partly reflects the low outcome prevalence. These calculations quantify a potential testing trade-off; they do not demonstrate an observed reduction in testing, cost-effectiveness or clinical benefit.

The study combines survey-weighted association estimates, fixed-sample comparisons with raw components and external validation without local model optimisation. Repeated primary-sampling-unit cross-validation and complementary performance measures reduce reliance on apparent AUC. Missingness diagnostics and a separate post-pandemic NHANES sample extend the robustness assessment.

Several limitations define the scope of inference. The cross-sectional design cannot establish temporality, incident diabetes risk or causality. FPG, 2-h glucose and HbA1c classify incompletely overlapping populations (5, 12). Without a consistently available Oral Glucose Tolerance Test (OGTT), we cannot identify isolated impaired glucose tolerance or exclude all cases of post-load diabetes. Restricting FPG and selecting HbA1c-defined cases may alter associations with adiposity-related indices, including possible overestimation; the direction and magnitude cannot be determined here. This selected outcome is not equivalent to all prediabetes.

The single local data source limits generalisation to other Chinese populations. Differences in case mix, time period, assays and clinical setting may influence both discrimination and calibration. Weighting and outcome imputation address only specified measured selection mechanisms and modelling assumptions; missing index components and unmeasured determinants of testing remain limitations. Linear candidate terms and data-derived Youden’s thresholds were evaluated without considering prospective testing pathways or economic outcomes. The non-replicated subgroup signals remain hypothesis-generating.

Any practical role is conditional. HbA1c is a standard diagnostic test included in WHO Essential Diagnostics List (50); this study demonstrates no advantage over direct HbA1c testing when that test is readily accessible. A staged approach might merit evaluation in health-examination programmes where fasting lipids and anthropometry are already recorded, but HbA1c capacity or reimbursement is limited. Ordering those measurements solely to calculate an index could instead increase the workload. A prospective comparison should include direct HbA1c testing and established non-laboratory risk assessment, such as the New Chinese Diabetes Risk Score (51), and measure missed cases, total costs and downstream outcomes. Until then, the benefit of index-guided testing remains theoretical.

## Conclusion

TyG–WHtR was associated with HbA1c-defined prediabetes among adults with FPG below 100 mg/dL, but it was not the strongest discriminator. Raw components led internal performance, whereas METS-IR led external AUC. These modest, setting-dependent differences support comparative validation rather than clinical implementation. Prospective studies must establish whether any index-guided pathway improves testing efficiency without unacceptable missed cases.

## Data Availability

Publicly available datasets were analyzed in this study. This data can be found here: https://www.cdc.gov/nchs/nhanes/.
