# Supplementary material for: External validation reveals setting-dependent performance of TyG-related indices for HbA1c-defined prediabetes
Source: Front Endocrinol (Lausanne). 2026 Oct 8;17:1961688. doi: 10.3389/fendo.2026.1961688 (PMC13686893; doi:10.3389/fendo.2026.1961688)
Supplement: Supplementary file 1 [file SupplementaryFile1.docx]

Supplementary information

# Supplementary methods

## Analysis chronology and provenance

The authors confirmed that the analysis code and an initial manuscript existed before 5 August 2026. Newly available records were added before submission and the existing workflow was rerun. The 5 August analysis plan documents that final workflow after earlier analyses had occurred. It is not evidence that the plan preceded every outcome analysis. The original wording has been corrected in the manuscript and in a dated plan amendment. Earlier-versus-updated result equivalence is an author-reported historical statement; a separately archived earlier dataset and complete original execution transcript were not available for direct comparison during this revision.

The retained results digest contains a generation timestamp of 5 August 2026 at 13:44:44 UTC, and the retained release-verification file contains a timestamp of 18:04:25 UTC on that date. File modification times and embedded timestamps can support the provenance of retained outputs but do not independently establish when the research questions were formulated. Execution logs generated during this revision record new work and must not be presented as August execution logs.

## Pooled NHANES weights and temporal differences

The seven derivation cycles each cover two years. Following the NCHS weighting tutorial, the fasting weight for the combined period is WTSAF2YR divided by seven. Ratios such as prevalence and weighted AUC are unchanged by a common positive scaling factor. Survey-weighted regression coefficients and design-based standard errors are likewise checked empirically under scaled and unscaled weights. Population totals change by a factor of seven. The sum of the pooled fasting weights is the average of the cycle-specific fasting-weight sums, not the total accumulated over 14 years.

Separate cycle estimates use each cycle’s original fasting weights. Age and sex are included in cycle-specific modified Poisson models; the same pooled survey-weighted TyG-WHtR mean and SD are used for every cycle. The pooled association model also includes survey cycle. A joint survey-adjusted Wald test evaluates TyG-WHtR-by-cycle interaction. These analyses distinguish a harmless change of weight scale from changes in the outcome or case mix across years. The pooled prevalence is interpreted as a period average.

## Model samples and added component models

Candidate availability counts require the outcome and only the predictors used by that candidate. Actual fitting and evaluation counts instead use the intersection of complete variables across the original candidate set. All original models therefore use 6,455 NHANES participants with 1,116 outcomes and 8,574 local participants with 928 outcomes. BMI and WHtR were available throughout these common samples. Their additional models were fitted only in NHANES and applied unchanged locally. No original frozen model or threshold was altered.

The original PSU grouping and seeds reproduce the five sets of 10 validation folds. All candidate models share every fold. Training and held-out sample sizes and outcomes are tabulated separately. Primary performance tables retain means across repeats, while curves, paired AUC differences, original thresholds and added IPW comparisons use averaged out-of-fold predictions. The two summaries differ slightly and should not be interchanged.

## Missing components and selection weighting

Among the 6,930 NHANES adults with an observed eligible outcome, 279 lack TyG-WHtR components. A total of 475 lack at least one variable required by the common prediction sample. These are distinct exclusions. Supplementary Table 9 shows means, SDs or proportions and variable-specific observed counts for complete and missing groups. NHANES descriptions use survey weights. Group SMDs use the square root of the mean of the two group variances as denominator. These comparisons describe measured differences only; a missing characteristic cannot be compared in participants for whom it is unobserved.

For NHANES common-record selection, a survey-weighted logistic model includes a three-degree-of-freedom natural spline of age, sex, cycle, FPG and race or ethnicity. Stabilised weights are the weighted common-record proportion divided by estimated inclusion probability. In cross-validation, each selection model is fitted only in training PSUs from the complete and incomplete outcome-eligible population. Models then use original survey weights, or survey weights multiplied by selection weights. Evaluation weighting and combined training-and-evaluation weighting are reported separately. No missing outcome is imputed for these AUC comparisons.

For local HbA1c availability, the target population consists of 12,721 adults meeting criteria ascertainable before HbA1c measurement, of whom 10,794 have measured HbA1c. Three observed participants are subsequently excluded for HbA1c-defined diabetes. The logistic availability model includes age, sex, enrolment year and FPG. Stabilised weights use the observed fraction divided by predicted availability. Truncation at the first and 99th percentiles is secondary. The same frozen predictions are assessed with and without these weights; there is no local coefficient refitting.

Balance checks compare the selected group with its full pre-selection target, using the target-population SD as a fixed denominator. For categorical variables the displayed summary is the maximum absolute SMD across levels; the full level-specific data are supplied separately. This estimand and denominator differ from the original available-versus-unavailable comparison whose maximum SMD was 0.047. The original 0.047 was calculated before weighting in a broader FPG <126 mg/dL population. Post-IPW balance and modest weights do not establish missing at random, remove selection on unmeasured characteristics or make the local cohort representative of China.

## Outcome imputation and departures from missing at random

This additional sensitivity retains adults with FPG <100 mg/dL, no known diabetes or pregnancy, and observed TyG-WHtR components, including those without HbA1c. NHANES additionally requires positive fasting survey weights and valid design variables. The imputation pools contain 6,679 NHANES adults, including 20 with missing HbA1c and eight with observed HbA1c-defined diabetes, and 11,322 local adults, including 1,705 with missing HbA1c and three with observed HbA1c-defined diabetes. Diabetes exclusions are reapplied after each completed dataset is classified.

Chained equations generate 50 completed datasets over 20 iterations. HbA1c, continuous missing covariates and kidney-function estimates use predictive mean matching; binary and multicategory covariates use logistic and polytomous regression. Predictors include TyG-WHtR, the association covariates and FPG, with log fasting weight added in NHANES. No index components are imputed. This is a separate sensitivity analysis; primary observed-outcome models remain unchanged. Index scaling is fixed to the original observed-outcome cohort-specific mean and SD.

To examine departures from MAR, only imputed HbA1c values are shifted by an additive delta from −0.5 to +0.5 percentage points in 0.1-point increments. Values are rounded to ten decimal places after shifting to avoid floating-point classification artefacts. HbA1c ≥6.5% is excluded and HbA1c 5.7–<6.5% defines the outcome in each scenario. Covariate imputations remain those generated under the original MAR model. Model 1 and Model 3 associations are estimated in each completed dataset and combined using Rubin’s rules with finite degrees of freedom.

This grid is an explicitly assumed pattern-mixture stress test, not an estimated mechanism or a validated plausible range. A uniform HbA1c shift does not cover all exposure-dependent nonignorable mechanisms. The exercise concerns robustness of association estimates, not external validation against imputed outcomes. Imputed outcomes are not used to claim discrimination or clinical utility. Chain summaries, logged events, and observed/imputed HbA1c distributions are retained with the analysis outputs.

## BMI and quartile descriptions

The original BMI groups are <25, 25–<30 and ≥30 kg/m² in both cohorts. The revision adds local Chinese categories <24, 24–<28 and ≥28 kg/m² as a separate post hoc sensitivity. Models adjust for age, sex and survey cycle or enrolment year. The original BMI interaction belongs to the original cohort-specific FDR family. The additional Chinese cut-point test is nominal and is not inserted into that family after inspection.

TyG-WHtR quartiles are calculated once within each cohort, using survey weights for NHANES, and applied unchanged in sex and age subgroups. NHANES prevalence uses design-based logit confidence intervals; local prevalence uses Wilson intervals. Counts are unweighted. Female and younger-adult proportions describe quartile composition, with survey weights in NHANES. These descriptive plots do not replace the adjusted interaction tests and do not establish that subgroup findings replicate.

The cohort-wide 25th, 50th and 75th percentile cut points were 3.9523474, 4.5388619 and 5.1895838 in NHANES and 3.6974754, 4.1154714 and 4.5579330 locally. Participants at a cut point belong to the lower quartile. These cut points were descriptive, not clinical thresholds.

## Retrospective testing trade-offs

Original model-specific probability thresholds were derived from NHANES out-of-fold predictions and applied at full precision to the local common sample. We count selected participants, detected outcomes and missed outcomes and express testing and missed-outcome counts per 1,000 eligible adults. All required predictor measurements are assumed to exist. No test prices, additional measurement costs, reimbursement, prospective testing behaviour, clinical outcomes or costs of missed cases were collected. The tabulation is a retrospective illustration and cannot establish cost-effectiveness or implementation benefit.

**Supplementary Table 1 Complete-case association estimates**

| **Cohort** | **Marker** | **Adjustment** | **n** | **Outcomes** | **PR (95% CI)** | **P** |
| --- | --- | --- | --- | --- | --- | --- |
| NHANES | TyG-WHtR | Model 1 | 6,651 | 1,152 | 1.298 (1.218 to 1.384) | 8.3e-16 |
| NHANES | TyG-WHtR | Model 2 | 5,638 | 951 | 1.270 (1.183 to 1.364) | 5.2e-11 |
| NHANES | TyG-WHtR | Model 3 | 4,609 | 816 | 1.250 (1.154 to 1.355) | 5.1e-08 |
| NHANES | TyG | Model 1 | 6,854 | 1,188 | 1.160 (1.075 to 1.252) | 0.00013 |
| NHANES | TyG | Model 2 | 5,755 | 972 | 1.128 (1.044 to 1.218) | 0.002 |
| NHANES | TyG | Model 3 | 4,699 | 835 | 1.119 (1.029 to 1.218) | 0.009 |
| NHANES | TyG-BMI | Model 1 | 6,791 | 1,180 | 1.303 (1.230 to 1.381) | 2.4e-19 |
| NHANES | TyG-BMI | Model 2 | 5,710 | 968 | 1.298 (1.221 to 1.379) | 4.8e-17 |
| NHANES | TyG-BMI | Model 3 | 4,666 | 832 | 1.276 (1.190 to 1.368) | 6.5e-12 |
| NHANES | TyG-WC | Model 1 | 6,657 | 1,153 | 1.284 (1.203 to 1.370) | 5.1e-14 |
| NHANES | TyG-WC | Model 2 | 5,643 | 951 | 1.278 (1.188 to 1.375) | 5.3e-11 |
| NHANES | TyG-WC | Model 3 | 4,613 | 816 | 1.260 (1.159 to 1.369) | 5.2e-08 |
| NHANES | METS-IR | Model 1 | 6,789 | 1,180 | 1.324 (1.248 to 1.405) | 1e-20 |
| NHANES | METS-IR | Model 2 | 5,708 | 968 | 1.301 (1.221 to 1.386) | 4.5e-16 |
| NHANES | METS-IR | Model 3 | 4,665 | 832 | 1.278 (1.191 to 1.372) | 1.2e-11 |
| NHANES | CMI | Model 1 | 6,649 | 1,152 | 1.112 (1.067 to 1.158) | 3.6e-07 |
| NHANES | CMI | Model 2 | 5,636 | 951 | 1.082 (1.035 to 1.131) | 0.00055 |
| NHANES | CMI | Model 3 | 4,608 | 816 | 1.074 (1.024 to 1.126) | 0.003 |
| NHANES | TG/HDL-C | Model 1 | 6,852 | 1,188 | 1.098 (1.053 to 1.145) | 1.1e-05 |
| NHANES | TG/HDL-C | Model 2 | 5,753 | 972 | 1.064 (1.014 to 1.115) | 0.011 |
| NHANES | TG/HDL-C | Model 3 | 4,698 | 835 | 1.057 (1.005 to 1.111) | 0.031 |
| NHANES | FPG | Model 1 | 6,930 | 1,206 | 1.252 (1.101 to 1.423) | 0.00058 |
| NHANES | FPG | Model 2 | 5,808 | 982 | 1.255 (1.079 to 1.459) | 0.003 |
| NHANES | FPG | Model 3 | 4,702 | 836 | 1.325 (1.207 to 1.455) | 3e-09 |
| NHANES | Triglycerides | Model 1 | 6,854 | 1,188 | 1.104 (1.048 to 1.162) | 0.00018 |
| NHANES | Triglycerides | Model 2 | 5,755 | 972 | 1.078 (1.017 to 1.142) | 0.011 |
| NHANES | Triglycerides | Model 3 | 4,699 | 835 | 1.075 (1.010 to 1.145) | 0.023 |
| NHANES | BMI | Model 1 | 6,863 | 1,196 | 1.291 (1.216 to 1.369) | 3.3e-17 |
| NHANES | BMI | Model 2 | 5,762 | 978 | 1.288 (1.210 to 1.370) | 1.7e-15 |
| NHANES | BMI | Model 3 | 4,669 | 833 | 1.264 (1.181 to 1.353) | 1.7e-11 |
| NHANES | WHtR | Model 1 | 6,720 | 1,166 | 1.285 (1.204 to 1.372) | 5.8e-14 |
| NHANES | WHtR | Model 2 | 5,689 | 960 | 1.259 (1.168 to 1.358) | 1.8e-09 |
| NHANES | WHtR | Model 3 | 4,612 | 817 | 1.237 (1.140 to 1.342) | 3.7e-07 |
| Local | TyG-WHtR | Model 1 | 9,614 | 1,036 | 1.480 (1.406 to 1.558) | 9.8e-51 |
| Local | TyG-WHtR | Model 2 | 7,355 | 795 | 1.506 (1.420 to 1.597) | 1.1e-42 |
| Local | TyG-WHtR | Model 3 | 5,972 | 637 | 1.491 (1.396 to 1.593) | 1.7e-32 |
| Local | TyG | Model 1 | 10,336 | 1,111 | 1.551 (1.470 to 1.636) | 5.1e-58 |
| Local | TyG | Model 2 | 7,890 | 857 | 1.538 (1.447 to 1.635) | 2.6e-43 |
| Local | TyG | Model 3 | 6,387 | 686 | 1.513 (1.415 to 1.619) | 1.9e-33 |
| Local | TyG-BMI | Model 1 | 9,898 | 1,060 | 1.558 (1.481 to 1.640) | 1.2e-65 |
| Local | TyG-BMI | Model 2 | 7,568 | 816 | 1.601 (1.512 to 1.695) | 7.5e-59 |
| Local | TyG-BMI | Model 3 | 6,140 | 654 | 1.597 (1.500 to 1.700) | 3e-48 |
| Local | TyG-WC | Model 1 | 9,823 | 1,060 | 1.540 (1.461 to 1.624) | 6.8e-58 |
| Local | TyG-WC | Model 2 | 7,510 | 816 | 1.573 (1.480 to 1.671) | 3.6e-48 |
| Local | TyG-WC | Model 3 | 6,098 | 654 | 1.564 (1.462 to 1.674) | 2.6e-38 |
| Local | METS-IR | Model 1 | 9,534 | 1,027 | 1.590 (1.515 to 1.668) | 4e-79 |
| Local | METS-IR | Model 2 | 7,297 | 792 | 1.627 (1.540 to 1.719) | 7.3e-68 |
| Local | METS-IR | Model 3 | 5,920 | 634 | 1.627 (1.533 to 1.727) | 9.7e-58 |
| Local | CMI | Model 1 | 9,256 | 1,003 | 1.355 (1.307 to 1.405) | 1.4e-60 |
| Local | CMI | Model 2 | 7,090 | 771 | 1.357 (1.299 to 1.418) | 4.4e-42 |
| Local | CMI | Model 3 | 5,757 | 617 | 1.333 (1.271 to 1.398) | 1.3e-32 |
| Local | TG/HDL-C | Model 1 | 9,952 | 1,077 | 1.359 (1.310 to 1.409) | 9.7e-62 |
| Local | TG/HDL-C | Model 2 | 7,605 | 832 | 1.358 (1.301 to 1.417) | 2.1e-44 |
| Local | TG/HDL-C | Model 3 | 6,156 | 665 | 1.338 (1.276 to 1.403) | 4.1e-33 |
| Local | FPG | Model 1 | 10,791 | 1,139 | 1.231 (1.159 to 1.308) | 1.6e-11 |
| Local | FPG | Model 2 | 8,223 | 880 | 1.228 (1.146 to 1.316) | 6.6e-09 |
| Local | FPG | Model 3 | 6,649 | 700 | 1.230 (1.137 to 1.329) | 2.1e-07 |
| Local | Triglycerides | Model 1 | 10,336 | 1,111 | 1.361 (1.311 to 1.413) | 3.9e-58 |
| Local | Triglycerides | Model 2 | 7,890 | 857 | 1.349 (1.291 to 1.409) | 4.8e-41 |
| Local | Triglycerides | Model 3 | 6,387 | 686 | 1.332 (1.268 to 1.400) | 3.3e-30 |
| Local | BMI | Model 1 | 10,336 | 1,087 | 1.408 (1.336 to 1.484) | 2.4e-37 |
| Local | BMI | Model 2 | 7,889 | 838 | 1.458 (1.375 to 1.546) | 1.4e-36 |
| Local | BMI | Model 3 | 6,392 | 667 | 1.467 (1.375 to 1.565) | 7.7e-31 |
| Local | WHtR | Model 1 | 10,026 | 1,061 | 1.303 (1.235 to 1.375) | 4.1e-22 |
| Local | WHtR | Model 2 | 7,656 | 815 | 1.331 (1.253 to 1.414) | 2.1e-20 |
| Local | WHtR | Model 3 | 6,209 | 648 | 1.328 (1.240 to 1.421) | 3e-16 |

Model 1 adjusts for age, sex and NHANES cycle or local enrolment year. Model 2 additionally adjusts for education, income-to-poverty ratio in NHANES or income category locally, smoking, alcohol use, and vigorous activity in NHANES or physical activity locally. Model 3 additionally adjusts for systolic blood pressure, hypertension, lipid-lowering medication and eGFR, with family history of diabetes included locally. PRs are per cohort-specific standard-deviation increase. NHANES uses design-based standard errors; local estimates use HC0 sandwich standard errors.

**Supplementary Table 2 Original internal candidate-model comparisons**

| **Model** | **ROC AUC** | **PR AUC** | **Brier** | **Calibration slope** | **AUC difference vs raw components (95% CI)** |
| --- | --- | --- | --- | --- | --- |
| Reference | 0.727 | 0.268 | 0.102 | 0.980 | -0.0192 (-0.0284 to -0.0100) |
| + FPG | 0.737 | 0.279 | 0.101 | 0.979 | -0.0093 (-0.0158 to -0.0034) |
| + TG | 0.727 | 0.267 | 0.102 | 0.977 | -0.0190 (-0.0287 to -0.0094) |
| + FPG + TG + WHtR | 0.746 | 0.289 | 0.100 | 0.974 | Reference |
| + TyG | 0.728 | 0.270 | 0.101 | 0.977 | -0.0180 (-0.0273 to -0.0089) |
| + TyG-BMI | 0.740 | 0.285 | 0.100 | 0.978 | -0.0061 (-0.0117 to -0.0002) |
| + TyG-WC | 0.736 | 0.281 | 0.101 | 0.977 | -0.0097 (-0.0153 to -0.0041) |
| + TyG-WHtR | 0.739 | 0.283 | 0.100 | 0.978 | -0.0073 (-0.0131 to -0.0016) |
| + METS-IR | 0.741 | 0.288 | 0.100 | 0.978 | -0.0053 (-0.0118 to 0.0011) |
| + CMI | 0.729 | 0.268 | 0.101 | 0.977 | -0.0173 (-0.0270 to -0.0076) |

All models use the same 6,455 participants and 1,116 outcomes. Reference includes age, sex, systolic blood pressure and smoking. Performance metrics are means across five repeats of 10-fold PSU-grouped cross-validation. AUC differences use averaged out-of-fold predictions and 1,000 cluster-bootstrap replicates.

**Supplementary Table 3 Original frozen external validation**

| **Model** | **AUC (95% CI)** | **PR AUC** | **Brier** | **Sensitivity** | **Specificity** | **PPV** | **NPV** |
| --- | --- | --- | --- | --- | --- | --- | --- |
| Reference | 0.672 (0.655 to 0.690) | 0.196 | 0.097 | 0.778 | 0.459 | 0.149 | 0.945 |
| + FPG | 0.682 (0.665 to 0.699) | 0.198 | 0.094 | 0.634 | 0.626 | 0.170 | 0.934 |
| + TG | 0.684 (0.666 to 0.701) | 0.204 | 0.097 | 0.810 | 0.429 | 0.147 | 0.949 |
| + FPG + TG + WHtR | 0.695 (0.678 to 0.712) | 0.212 | 0.092 | 0.555 | 0.702 | 0.184 | 0.929 |
| + TyG | 0.693 (0.676 to 0.710) | 0.211 | 0.096 | 0.816 | 0.442 | 0.151 | 0.952 |
| + TyG-BMI | 0.704 (0.687 to 0.721) | 0.223 | 0.093 | 0.805 | 0.471 | 0.156 | 0.952 |
| + TyG-WC | 0.703 (0.686 to 0.719) | 0.221 | 0.092 | 0.765 | 0.526 | 0.164 | 0.949 |
| + TyG-WHtR | 0.703 (0.686 to 0.720) | 0.222 | 0.092 | 0.759 | 0.534 | 0.165 | 0.948 |
| + METS-IR | 0.710 (0.693 to 0.727) | 0.228 | 0.093 | 0.798 | 0.500 | 0.162 | 0.953 |
| + CMI | 0.691 (0.673 to 0.708) | 0.211 | 0.096 | 0.814 | 0.443 | 0.150 | 0.951 |

All models use the same 8,574 participants and 928 outcomes. Models and model-specific probability thresholds are frozen from NHANES. AUC intervals use DeLong methods. PPV and NPV depend on outcome prevalence. Reference includes age, sex, systolic blood pressure and smoking.

**Supplementary Table 4 Original subgroup associations and interactions**

| **Cohort** | **Subgroup** | **Level** | **n / outcomes** | **PR (95% CI)** | **Interaction P** | **FDR P** |
| --- | --- | --- | --- | --- | --- | --- |
| NHANES | sex | Male | 2,744 / 469 | 1.069 (0.931 to 1.228) | 0.00038 | 0.002 |
| NHANES | sex | Female | 3,907 / 683 | 1.416 (1.314 to 1.526) | 0.00038 | 0.002 |
| NHANES | age group | <50 | 4,395 / 452 | 1.453 (1.330 to 1.588) | 0.009 | 0.017 |
| NHANES | age group | >=50 | 2,256 / 700 | 1.223 (1.122 to 1.333) | 0.009 | 0.017 |
| NHANES | bmi group | <25 | 2,673 / 350 | 1.388 (1.119 to 1.720) | 0.096 | 0.116 |
| NHANES | bmi group | 25-<30 | 2,147 / 382 | 1.105 (0.867 to 1.407) | 0.096 | 0.116 |
| NHANES | bmi group | >=30 | 1,827 / 419 | 1.157 (1.007 to 1.329) | 0.096 | 0.116 |
| NHANES | hypertension | No | 5,078 / 696 | 1.357 (1.236 to 1.490) | 0.019 | 0.029 |
| NHANES | hypertension | Yes | 1,566 / 456 | 1.132 (1.011 to 1.267) | 0.019 | 0.029 |
| NHANES | lipid lowering | No | 4,956 / 768 | 1.357 (1.254 to 1.467) | 0.007 | 0.017 |
| NHANES | lipid lowering | Yes | 610 / 238 | 1.066 (0.922 to 1.233) | 0.007 | 0.017 |
| NHANES | race ethnicity | Mexican American | 943 / 152 | 1.297 (1.072 to 1.570) | 0.643 | 0.643 |
| NHANES | race ethnicity | Non-Hispanic Black | 1,431 / 429 | 1.278 (1.148 to 1.424) | 0.643 | 0.643 |
| NHANES | race ethnicity | Non-Hispanic White | 2,887 / 365 | 1.332 (1.201 to 1.477) | 0.643 | 0.643 |
| NHANES | race ethnicity | Other Hispanic | 639 / 98 | 1.348 (1.057 to 1.718) | 0.643 | 0.643 |
| NHANES | race ethnicity | Other or multiracial | 751 / 108 | 1.267 (1.012 to 1.585) | 0.643 | 0.643 |
| Local | sex | Male | 4,671 / 496 | 1.512 (1.403 to 1.629) | 0.438 | 0.528 |
| Local | sex | Female | 4,943 / 540 | 1.453 (1.354 to 1.559) | 0.438 | 0.528 |
| Local | age group | <50 | 4,794 / 281 | 1.644 (1.483 to 1.823) | 0.028 | 0.138 |
| Local | age group | >=50 | 4,820 / 755 | 1.437 (1.354 to 1.525) | 0.028 | 0.138 |
| Local | bmi group | <25 | 5,610 / 459 | 1.480 (1.339 to 1.635) | 0.498 | 0.528 |
| Local | bmi group | 25-<30 | 3,350 / 457 | 1.384 (1.248 to 1.535) | 0.498 | 0.528 |
| Local | bmi group | >=30 | 448 / 97 | 1.312 (1.081 to 1.593) | 0.498 | 0.528 |
| Local | hypertension | No | 8,689 / 899 | 1.491 (1.410 to 1.576) | 0.207 | 0.518 |
| Local | hypertension | Yes | 744 / 118 | 1.345 (1.166 to 1.552) | 0.207 | 0.518 |
| Local | lipid lowering | No | 8,741 / 932 | 1.472 (1.394 to 1.555) | 0.528 | 0.528 |
| Local | lipid lowering | Yes | 375 / 44 | 1.424 (1.139 to 1.781) | 0.528 | 0.528 |

PRs are per cohort-specific SD. Models adjust for age, sex and cycle/year, except that the stratifying sex or age term is omitted in the corresponding analysis. Interaction P is from the original cohort-specific model; FDR uses the original interaction family separately by cohort. These analyses are hypothesis-generating.

**Supplementary Table 5 Original sensitivity analyses**

| **Cohort** | **Analysis** | **n** | **Outcomes** | **Effect** | **Estimate (95% CI)** | **P** |
| --- | --- | --- | --- | --- | --- | --- |
| NHANES | Primary ADA FPG <100 | 6,651 | 1,152 | PR | 1.298 (1.218 to 1.384) | 8.3e-16 |
| NHANES | WHO-normal FPG <110 | 10,455 | 2,455 | PR | 1.358 (1.299 to 1.420) | 1.4e-41 |
| NHANES | FPG <95 | 4,048 | 560 | PR | 1.310 (1.203 to 1.428) | 6.6e-10 |
| NHANES | Exclude lipid-lowering therapy | 4,956 | 768 | PR | 1.358 (1.255 to 1.470) | 2.7e-14 |
| NHANES | eGFR >=30 | 6,595 | 1,138 | PR | 1.299 (1.218 to 1.386) | 2e-15 |
| NHANES | Logistic sensitivity | 6,651 | 1,152 | OR | 1.387 (1.276 to 1.507) | 1.2e-14 |
| Local | Primary ADA FPG <100 | 9,614 | 1,036 | PR | 1.480 (1.406 to 1.558) | 9.8e-51 |
| Local | WHO-normal FPG <110 | 11,043 | 1,276 | PR | 1.482 (1.415 to 1.552) | 1.6e-62 |
| Local | FPG <95 | 8,217 | 826 | PR | 1.481 (1.396 to 1.570) | 4e-39 |
| Local | Exclude lipid-lowering therapy | 8,741 | 932 | PR | 1.471 (1.393 to 1.553) | 7.4e-44 |
| Local | eGFR >=30 | 9,177 | 995 | PR | 1.474 (1.399 to 1.554) | 1.5e-47 |
| Local | Enrollment 2020-2023 | 4,880 | 544 | PR | 1.458 (1.362 to 1.561) | 1.6e-27 |
| Local | Enrollment 2024-2026 | 4,734 | 492 | PR | 1.504 (1.392 to 1.626) | 9.3e-25 |
| Local | Logistic sensitivity | 9,614 | 1,036 | OR | 1.611 (1.508 to 1.721) | 1.8e-45 |
| Local | HbA1c availability IPW | 9,614 | 1,036 | PR | 1.477 (1.403 to 1.554) | 5.2e-50 |
| Local | HbA1c availability IPW truncated 1/99 | 9,614 | 1,036 | PR | 1.477 (1.403 to 1.554) | 5.2e-50 |
| NHANES 2021-2023 | Temporal sensitivity | 1,146 | 196 | PR | 1.510 (1.353 to 1.685) | 1.8e-13 |

Models adjust for age, sex and survey cycle/enrolment year, with no cycle term in the separate 2021–2023 NHANES sample. Estimates use the original sensitivity-specific scaling. Logistic analyses report odds ratios; the others report prevalence ratios. Availability weighting is specific to the local cohort.

**Supplementary Table 6a Correlations with log HOMA-IR**

| **Cohort** | **Marker** | **n** | **Pearson correlation** | **Spearman correlation** |
| --- | --- | --- | --- | --- |
| NHANES | TyG | 4,070 | 0.377 | 0.378 |
| NHANES | TyG-BMI | 4,029 | 0.605 | 0.607 |
| NHANES | TyG-WC | 3,964 | 0.592 | 0.577 |
| NHANES | TyG-WHtR | 3,961 | 0.591 | 0.589 |
| NHANES | METS-IR | 4,028 | 0.613 | 0.612 |
| NHANES | CMI | 3,960 | 0.329 | 0.485 |
| Local | TyG | 6,884 | 0.312 | 0.300 |
| Local | TyG-BMI | 6,603 | 0.412 | 0.394 |
| Local | TyG-WC | 6,556 | 0.356 | 0.347 |
| Local | TyG-WHtR | 6,426 | 0.360 | 0.350 |
| Local | METS-IR | 6,367 | 0.411 | 0.395 |
| Local | CMI | 6,193 | 0.283 | 0.311 |

Pearson correlations use fasting survey weights in NHANES and unit weights locally. Spearman correlations are unweighted. HOMA-IR and TyG share FPG, so these are convergent metabolic results, not mechanistic validation.

**Supplementary Table 6b Adjusted association with HOMA-IR**

| **Cohort** | **n** | **Geometric mean ratio (95% CI)** | **P** |
| --- | --- | --- | --- |
| NHANES | 3,961 | 1.551 (1.513 to 1.589) | 6.1e-271 |
| Local | 6,426 | 1.205 (1.191 to 1.219) | 5.9e-215 |

Estimates describe log HOMA-IR per cohort-specific SD in TyG-WHtR, adjusted for age and sex plus survey cycle or enrolment year.

**Supplementary Table 7 Secondary intercept-only recalibration**

| **Model** | **ROC AUC** | **PR AUC** | **Brier** | **Calibration intercept** | **Calibration slope** |
| --- | --- | --- | --- | --- | --- |
| Reference | 0.672 | 0.196 | 0.093 | -0.120 | 0.939 |
| + FPG | 0.682 | 0.198 | 0.093 | -0.450 | 0.764 |
| + TG | 0.684 | 0.204 | 0.092 | 0.030 | 1.015 |
| + FPG + TG + WHtR | 0.695 | 0.212 | 0.092 | -0.322 | 0.830 |
| + TyG | 0.693 | 0.211 | 0.092 | 0.140 | 1.072 |
| + TyG-BMI | 0.704 | 0.223 | 0.091 | 0.058 | 1.030 |
| + TyG-WC | 0.703 | 0.221 | 0.091 | 0.157 | 1.081 |
| + TyG-WHtR | 0.703 | 0.222 | 0.091 | 0.156 | 1.081 |
| + METS-IR | 0.710 | 0.228 | 0.091 | 0.098 | 1.052 |
| + CMI | 0.691 | 0.211 | 0.092 | 0.100 | 1.051 |

Local intercept-only recalibration is a secondary analysis on the same 8,574 participants. It does not replace primary frozen external validation. The calibration intercept shown here is from the joint intercept-and-slope model, not calibration-in-the-large.

**Supplementary Table 8a Candidate availability and actual model samples**

| **Cohort** | **Model** | **Candidate available n / outcomes** | **Actual common n / outcomes** | **Parameters** | **Status** |
| --- | --- | --- | --- | --- | --- |
| NHANES | Reference | 6,686 / 1,161 | 6,455 / 1,116 | 6 | Original |
| Local | Reference | 10,194 / 1,077 | 8,574 / 928 | 6 | Original |
| NHANES | + FPG | 6,686 / 1,161 | 6,455 / 1,116 | 7 | Original |
| Local | + FPG | 10,194 / 1,077 | 8,574 / 928 | 7 | Original |
| NHANES | + TG | 6,616 / 1,145 | 6,455 / 1,116 | 7 | Original |
| Local | + TG | 9,774 / 1,053 | 8,574 / 928 | 7 | Original |
| NHANES | + FPG + TG + WHtR | 6,461 / 1,117 | 6,455 / 1,116 | 9 | Original |
| Local | + FPG + TG + WHtR | 9,096 / 981 | 8,574 / 928 | 9 | Original |
| NHANES | + TyG | 6,616 / 1,145 | 6,455 / 1,116 | 7 | Original |
| Local | + TyG | 9,774 / 1,053 | 8,574 / 928 | 7 | Original |
| NHANES | + TyG-BMI | 6,569 / 1,139 | 6,455 / 1,116 | 7 | Original |
| Local | + TyG-BMI | 9,363 / 1,003 | 8,574 / 928 | 7 | Original |
| NHANES | + TyG-WC | 6,467 / 1,118 | 6,455 / 1,116 | 7 | Original |
| Local | + TyG-WC | 9,293 / 1,004 | 8,574 / 928 | 7 | Original |
| NHANES | + TyG-WHtR | 6,461 / 1,117 | 6,455 / 1,116 | 7 | Original |
| Local | + TyG-WHtR | 9,096 / 981 | 8,574 / 928 | 7 | Original |
| NHANES | + METS-IR | 6,567 / 1,139 | 6,455 / 1,116 | 7 | Original |
| Local | + METS-IR | 9,018 / 972 | 8,574 / 928 | 7 | Original |
| NHANES | + CMI | 6,459 / 1,117 | 6,455 / 1,116 | 7 | Original |
| Local | + CMI | 8,758 / 950 | 8,574 / 928 | 7 | Original |
| NHANES | + BMI | 6,637 / 1,154 | 6,455 / 1,116 | 7 | Added secondary |
| Local | + BMI | 9,768 / 1,026 | 8,574 / 928 | 7 | Added secondary |
| NHANES | + WHtR | 6,527 / 1,130 | 6,455 / 1,116 | 7 | Added secondary |
| Local | + WHtR | 9,477 / 1,002 | 8,574 / 928 | 7 | Added secondary |

Candidate availability counts require only the variables used by that candidate; they are not different fitting samples. Every model was fitted in the fixed NHANES common sample and assessed in the fixed local common sample. Reference includes age, sex, systolic blood pressure and smoking. Parameters include the intercept. BMI and WHtR were added during revision without changing common-sample membership.

**Supplementary Table 8b Training and held-out samples in each shared fold**

| **Repeat** | **Fold** | **Training n** | **Training outcomes** | **Held-out n** | **Held-out outcomes** |
| --- | --- | --- | --- | --- | --- |
| 1 | 1 | 5,777 | 989 | 678 | 127 |
| 1 | 2 | 5,701 | 979 | 754 | 137 |
| 1 | 3 | 5,771 | 1,004 | 684 | 112 |
| 1 | 4 | 5,816 | 1,005 | 639 | 111 |
| 1 | 5 | 5,864 | 1,007 | 591 | 109 |
| 1 | 6 | 5,815 | 1,007 | 640 | 109 |
| 1 | 7 | 5,755 | 1,002 | 700 | 114 |
| 1 | 8 | 5,855 | 1,017 | 600 | 99 |
| 1 | 9 | 5,885 | 1,021 | 570 | 95 |
| 1 | 10 | 5,856 | 1,013 | 599 | 103 |
| 2 | 1 | 5,738 | 986 | 717 | 130 |
| 2 | 2 | 5,687 | 987 | 768 | 129 |
| 2 | 3 | 5,729 | 994 | 726 | 122 |
| 2 | 4 | 5,769 | 995 | 686 | 121 |
| 2 | 5 | 5,833 | 1,013 | 622 | 103 |
| 2 | 6 | 5,923 | 1,019 | 532 | 97 |
| 2 | 7 | 5,855 | 1,016 | 600 | 100 |
| 2 | 8 | 5,848 | 1,010 | 607 | 106 |
| 2 | 9 | 5,878 | 1,015 | 577 | 101 |
| 2 | 10 | 5,835 | 1,009 | 620 | 107 |
| 3 | 1 | 5,654 | 982 | 801 | 134 |
| 3 | 2 | 5,711 | 978 | 744 | 138 |
| 3 | 3 | 5,848 | 1,010 | 607 | 106 |
| 3 | 4 | 5,760 | 1,000 | 695 | 116 |
| 3 | 5 | 5,799 | 1,006 | 656 | 110 |
| 3 | 6 | 5,790 | 1,000 | 665 | 116 |
| 3 | 7 | 5,864 | 1,014 | 591 | 102 |
| 3 | 8 | 5,862 | 1,010 | 593 | 106 |
| 3 | 9 | 5,900 | 1,017 | 555 | 99 |
| 3 | 10 | 5,907 | 1,027 | 548 | 89 |
| 4 | 1 | 5,705 | 988 | 750 | 128 |
| 4 | 2 | 5,823 | 1,002 | 632 | 114 |
| 4 | 3 | 5,818 | 1,008 | 637 | 108 |
| 4 | 4 | 5,781 | 995 | 674 | 121 |
| 4 | 5 | 5,829 | 1,019 | 626 | 97 |
| 4 | 6 | 5,751 | 998 | 704 | 118 |
| 4 | 7 | 5,813 | 997 | 642 | 119 |
| 4 | 8 | 5,849 | 1,002 | 606 | 114 |
| 4 | 9 | 5,816 | 1,013 | 639 | 103 |
| 4 | 10 | 5,910 | 1,022 | 545 | 94 |
| 5 | 1 | 5,784 | 997 | 671 | 119 |
| 5 | 2 | 5,762 | 992 | 693 | 124 |
| 5 | 3 | 5,658 | 983 | 797 | 133 |
| 5 | 4 | 5,826 | 1,005 | 629 | 111 |
| 5 | 5 | 5,789 | 1,004 | 666 | 112 |
| 5 | 6 | 5,857 | 1,014 | 598 | 102 |
| 5 | 7 | 5,753 | 997 | 702 | 119 |
| 5 | 8 | 5,865 | 1,015 | 590 | 101 |
| 5 | 9 | 5,883 | 1,017 | 572 | 99 |
| 5 | 10 | 5,918 | 1,020 | 537 | 96 |

The same folds are used by all 12 candidate models. Entire PSUs stay in one fold. These are unweighted participant and outcome counts.

**Supplementary Table 9a NHANES 2005-2018: observed versus missing TyG-WHtR components**

| **Characteristic** | **Complete (n=6,651)** | **Missing (n=279)** | **Observed values complete / missing** | **SMD** |
| --- | --- | --- | --- | --- |
| Age | 41.97 (15.79) | 47.29 (19.36) | 6,651 / 279 | -0.301 |
| Female | 58.8% | 65.4% | 6,651 / 279 | -0.138 |
| BMI | 27.09 (6.02) | 27.76 (8.24) | 6,647 / 216 | -0.093 |
| Waist circumference | 93.24 (14.71) | 92.44 (18.25) | 6,651 / 75 | 0.049 |
| Height | 168.34 (9.77) | 166.06 (10.36) | 6,651 / 217 | 0.226 |
| FPG | 91.80 (5.73) | 91.88 (5.53) | 6,651 / 279 | -0.015 |
| Triglycerides | 107.04 (78.48) | 102.63 (78.25) | 6,651 / 203 | 0.056 |
| HDL-C | 57.36 (16.32) | 59.29 (17.39) | 6,649 / 210 | -0.114 |
| Systolic blood pressure | 117.09 (15.12) | 120.36 (23.23) | 6,465 / 225 | -0.167 |
| HbA1c | 5.27 (0.34) | 5.29 (0.35) | 6,651 / 279 | -0.052 |
| HbA1c-defined prediabetes | 12.7% | 13.5% | 6,651 / 279 | -0.024 |
| Smoking Never | 58.4% | 56.6% | 6,646 / 278 | 0.036 |
| Smoking Former | 20.2% | 22.3% | 6,646 / 278 | -0.051 |
| Smoking Current | 21.4% | 21.1% | 6,646 / 278 | 0.008 |
| Hypertension | 20.6% | 36.3% | 6,644 / 277 | -0.353 |
| Lipid-lowering medication | 9.8% | 11.1% | 5,566 / 240 | -0.040 |
| Survey cycle 2005-2006 | 15.6% | 14.1% | 6,651 / 279 | 0.044 |
| Survey cycle 2007-2008 | 13.2% | 14.7% | 6,651 / 279 | -0.042 |
| Survey cycle 2009-2010 | 15.3% | 12.1% | 6,651 / 279 | 0.093 |
| Survey cycle 2011-2012 | 15.4% | 14.0% | 6,651 / 279 | 0.038 |
| Survey cycle 2013-2014 | 16.6% | 14.8% | 6,651 / 279 | 0.048 |
| Survey cycle 2015-2016 | 12.8% | 10.2% | 6,651 / 279 | 0.081 |
| Survey cycle 2017-2018 | 11.1% | 20.1% | 6,651 / 279 | -0.250 |
| Race or ethnicity Mexican American | 7.7% | 4.5% | 6,651 / 279 | 0.132 |
| Race or ethnicity Non-Hispanic Black | 12.2% | 20.6% | 6,651 / 279 | -0.228 |
| Race or ethnicity Non-Hispanic White | 67.3% | 60.7% | 6,651 / 279 | 0.138 |
| Race or ethnicity Other Hispanic | 5.7% | 6.1% | 6,651 / 279 | -0.017 |
| Race or ethnicity Other or multiracial | 7.1% | 8.1% | 6,651 / 279 | -0.035 |

Continuous values are means (SD); categorical values are percentages. Units are years for age, kg/m² for BMI, cm for height/waist, mg/dL for glucose and lipids, mmHg for systolic pressure, and % for HbA1c. NHANES summaries use survey weights. SMD is complete minus missing, divided by the square root of the average group variances. Each characteristic uses its observed values only; variable-specific observed counts disclose remaining missingness. Missing-component counts can overlap.

**Supplementary Table 9b NHANES 2005-2018: observed versus missing common-model variables**

| **Characteristic** | **Complete (n=6,455)** | **Missing (n=475)** | **Observed values complete / missing** | **SMD** |
| --- | --- | --- | --- | --- |
| Age | 42.03 (15.83) | 44.12 (17.81) | 6,455 / 475 | -0.124 |
| Female | 58.6% | 66.2% | 6,455 / 475 | -0.158 |
| BMI | 27.08 (6.00) | 27.56 (7.57) | 6,455 / 408 | -0.070 |
| Waist circumference | 93.24 (14.68) | 93.10 (16.74) | 6,455 / 271 | 0.009 |
| Height | 168.36 (9.78) | 166.74 (9.84) | 6,455 / 413 | 0.165 |
| FPG | 91.83 (5.65) | 91.34 (6.70) | 6,455 / 475 | 0.078 |
| Triglycerides | 107.34 (79.11) | 98.90 (64.12) | 6,455 / 399 | 0.117 |
| HDL-C | 57.36 (16.29) | 58.33 (17.39) | 6,455 / 404 | -0.058 |
| Systolic blood pressure | 117.09 (15.12) | 120.30 (22.96) | 6,455 / 235 | -0.165 |
| HbA1c | 5.27 (0.34) | 5.29 (0.37) | 6,455 / 475 | -0.040 |
| HbA1c-defined prediabetes | 12.6% | 15.0% | 6,455 / 475 | -0.070 |
| Smoking Never | 58.2% | 59.6% | 6,455 / 469 | -0.029 |
| Smoking Former | 20.4% | 18.1% | 6,455 / 469 | 0.060 |
| Smoking Current | 21.3% | 22.3% | 6,455 / 469 | -0.024 |
| Hypertension | 20.7% | 29.0% | 6,448 / 473 | -0.192 |
| Lipid-lowering medication | 9.9% | 9.5% | 5,401 / 405 | 0.013 |
| Survey cycle 2005-2006 | 15.7% | 12.9% | 6,455 / 475 | 0.081 |
| Survey cycle 2007-2008 | 13.3% | 12.7% | 6,455 / 475 | 0.017 |
| Survey cycle 2009-2010 | 15.3% | 13.4% | 6,455 / 475 | 0.055 |
| Survey cycle 2011-2012 | 15.2% | 16.9% | 6,455 / 475 | -0.046 |
| Survey cycle 2013-2014 | 16.7% | 13.4% | 6,455 / 475 | 0.094 |
| Survey cycle 2015-2016 | 12.8% | 11.6% | 6,455 / 475 | 0.038 |
| Survey cycle 2017-2018 | 10.9% | 19.1% | 6,455 / 475 | -0.232 |
| Race or ethnicity Mexican American | 7.6% | 6.3% | 6,455 / 475 | 0.054 |
| Race or ethnicity Non-Hispanic Black | 12.0% | 19.8% | 6,455 / 475 | -0.213 |
| Race or ethnicity Non-Hispanic White | 67.5% | 60.3% | 6,455 / 475 | 0.150 |
| Race or ethnicity Other Hispanic | 5.7% | 5.9% | 6,455 / 475 | -0.010 |
| Race or ethnicity Other or multiracial | 7.1% | 7.7% | 6,455 / 475 | -0.020 |

Continuous values are means (SD); categorical values are percentages. Units are years for age, kg/m² for BMI, cm for height/waist, mg/dL for glucose and lipids, mmHg for systolic pressure, and % for HbA1c. NHANES summaries use survey weights. SMD is complete minus missing, divided by the square root of the average group variances. Each characteristic uses its observed values only; variable-specific observed counts disclose remaining missingness. Missing-component counts can overlap.

**Supplementary Table 9c Local cohort: observed versus missing TyG-WHtR components**

| **Characteristic** | **Complete (n=9,614)** | **Missing (n=1,177)** | **Observed values complete / missing** | **SMD** |
| --- | --- | --- | --- | --- |
| Age | 49.61 (12.46) | 49.39 (12.59) | 9,614 / 1,177 | 0.017 |
| Female | 51.4% | 48.3% | 9,614 / 1,177 | 0.063 |
| BMI | 24.07 (3.48) | 24.04 (3.40) | 9,408 / 928 | 0.007 |
| Waist circumference | 79.11 (10.49) | 78.95 (10.24) | 9,614 / 631 | 0.015 |
| Height | 162.80 (7.81) | 162.96 (7.59) | 9,614 / 945 | -0.020 |
| FPG | 84.62 (9.41) | 84.56 (9.45) | 9,614 / 1,177 | 0.006 |
| Triglycerides | 129.31 (65.17) | 131.65 (67.07) | 9,614 / 722 | -0.035 |
| HDL-C | 50.68 (13.26) | 51.24 (13.55) | 9,256 / 1,132 | -0.042 |
| Systolic blood pressure | 120.05 (14.97) | 119.73 (14.60) | 9,369 / 1,134 | 0.022 |
| HbA1c | 5.24 (0.36) | 5.24 (0.35) | 9,614 / 1,177 | 0.012 |
| HbA1c-defined prediabetes | 10.8% | 8.8% | 9,614 / 1,177 | 0.068 |
| Smoking Never | 69.7% | 67.9% | 9,336 / 1,140 | 0.040 |
| Smoking Former | 6.8% | 6.9% | 9,336 / 1,140 | -0.006 |
| Smoking Current | 23.5% | 25.2% | 9,336 / 1,140 | -0.039 |
| Hypertension | 7.9% | 7.6% | 9,433 / 1,158 | 0.011 |
| Lipid-lowering medication | 4.1% | 4.5% | 9,116 / 1,118 | -0.018 |
| Enrolment year 2020 | 10.4% | 16.8% | 9,614 / 1,177 | -0.188 |
| Enrolment year 2021 | 11.7% | 18.3% | 9,614 / 1,177 | -0.185 |
| Enrolment year 2022 | 13.2% | 16.7% | 9,614 / 1,177 | -0.098 |
| Enrolment year 2023 | 15.4% | 15.9% | 9,614 / 1,177 | -0.013 |
| Enrolment year 2024 | 16.5% | 12.1% | 9,614 / 1,177 | 0.126 |
| Enrolment year 2025 | 18.8% | 13.0% | 9,614 / 1,177 | 0.158 |
| Enrolment year 2026 | 13.9% | 7.1% | 9,614 / 1,177 | 0.223 |

Continuous values are means (SD); categorical values are percentages. Units are years for age, kg/m² for BMI, cm for height/waist, mg/dL for glucose and lipids, mmHg for systolic pressure, and % for HbA1c. NHANES summaries use survey weights. SMD is complete minus missing, divided by the square root of the average group variances. Each characteristic uses its observed values only; variable-specific observed counts disclose remaining missingness. Missing-component counts can overlap.

**Supplementary Table 10a Constant weight rescaling check**

| **Weights** | **Prevalence (95% CI)** | **PR (95% CI)** | **TyG-WHtR-model AUC** | **Fasting weight sum** |
| --- | --- | --- | --- | --- |
| Pooled WTSAF2YR / 7 | 12.72% (11.72 to 13.72) | 1.298 (1.218 to 1.384) | 0.738704 | 257,906,230 |
| Unscaled WTSAF2YR | 12.72% (11.72 to 13.72) | 1.298 (1.218 to 1.384) | 0.738704 | 1,805,343,614 |

The prevalence sample is n=6,930 with 1,206 outcomes; the association sample is n=6,651 with 1,152 outcomes. Model 1 adjusts for age, sex and cycle using one common survey-weighted SD. AUC uses the same averaged out-of-fold predictions and common sample. Dividing all weights by seven changes population totals, but not these ratio estimates or regression coefficients. This is not a test that selection bias or time trends are absent.

**Supplementary Table 10b Cycle-specific estimates with original two-year fasting weights**

| **Cycle** | **Eligible n / outcomes** | **Association n / outcomes** | **Prevalence (95% CI)** | **PR (95% CI)** |
| --- | --- | --- | --- | --- |
| 2005-2006 | 923 / 105 | 886 / 100 | 7.8% (6.5 to 9.1) | 1.465 (1.208 to 1.778) |
| 2007-2008 | 938 / 161 | 902 / 153 | 12.4% (10.1 to 14.6) | 1.183 (0.957 to 1.464) |
| 2009-2010 | 1,223 / 251 | 1,190 / 244 | 16.3% (14.1 to 18.5) | 1.279 (1.084 to 1.509) |
| 2011-2012 | 1,089 / 202 | 1,043 / 194 | 14.8% (11.6 to 18.0) | 1.146 (1.002 to 1.310) |
| 2013-2014 | 1,172 / 210 | 1,128 / 203 | 12.9% (10.6 to 15.2) | 1.333 (1.205 to 1.474) |
| 2015-2016 | 840 / 154 | 809 / 147 | 12.9% (10.1 to 15.7) | 1.346 (1.105 to 1.641) |
| 2017-2018 | 745 / 123 | 693 / 111 | 11.7% (7.9 to 15.5) | 1.538 (1.273 to 1.857) |

Associations adjust for age and sex, using the same pooled NHANES SD in every cycle. Individual-cycle estimates have different case mixes and lower precision. The pooled TyG-WHtR-by-cycle interaction P is 0.193. Weight scaling preserves each cycle’s relative contribution; temporal heterogeneity must be assessed separately.

**Supplementary Table 11 BMI-stratified TyG-WHtR associations**

| **Cohort / cut points** | **BMI (kg/m²)** | **n / outcomes** | **PR (95% CI)** | **Interaction P** | **FDR P** |
| --- | --- | --- | --- | --- | --- |
| NHANES | <25 | 2,673 / 350 | 1.388 (1.119 to 1.720) | 0.096 | 0.116 |
| NHANES | 25-<30 | 2,147 / 382 | 1.105 (0.867 to 1.407) | 0.096 | 0.116 |
| NHANES | >=30 | 1,827 / 419 | 1.157 (1.007 to 1.329) | 0.096 | 0.116 |
| Local | <25 | 5,610 / 459 | 1.480 (1.339 to 1.635) | 0.498 | 0.528 |
| Local | 25-<30 | 3,350 / 457 | 1.384 (1.248 to 1.535) | 0.498 | 0.528 |
| Local | >=30 | 448 / 97 | 1.312 (1.081 to 1.593) | 0.498 | 0.528 |
| Local Chinese cut points | <24 | 4,588 / 353 | 1.485 (1.319 to 1.672) | 0.435 | NA |
| Local Chinese cut points | 24-<28 | 3,579 / 433 | 1.415 (1.264 to 1.583) | 0.435 | NA |
| Local Chinese cut points | >=28 | 1,241 / 227 | 1.320 (1.161 to 1.500) | 0.435 | NA |

Models adjust for age, sex and cycle or enrolment year. The index uses the overall cohort-specific SD, not a within-stratum SD. International thresholds were part of the original subgroup family; Chinese thresholds were added post hoc and are not included in that FDR family. NA indicates that no adjusted P was assigned to the additional analysis.

**Supplementary Table 12a Measured balance relative to the pre-selection target**

| **Selection mechanism** | **Characteristic** | **Maximum absolute SMD before** | **Maximum absolute SMD after IPW** |
| --- | --- | --- | --- |
| Local HbA1c | Age | 0.0013 | 0.0004 |
| Local HbA1c | Female | 0.0063 | 0.0002 |
| Local HbA1c | Enrolment year | 0.0394 | 0.0001 |
| Local HbA1c | FPG | 0.0005 | 0.0004 |
| Local HbA1c | BMI | 0.0037 | 0.0038 |
| Local HbA1c | Waist circumference | 0.0033 | 0.0017 |
| Local HbA1c | Triglycerides | 0.0010 | 0.0007 |
| Local HbA1c | HDL-C | 0.0026 | 0.0039 |
| Local HbA1c | Systolic blood pressure | 0.0059 | 0.0048 |
| Local HbA1c | Smoking | 0.0093 | 0.0058 |
| Local HbA1c | Hypertension | 0.0008 | 0.0001 |
| Local HbA1c | Lipid-lowering medication | 0.0034 | 0.0032 |
| Local HbA1c | Family history of diabetes | 0.0008 | 0.0003 |
| NHANES common sample | Age | 0.0076 | 0.0001 |
| NHANES common sample | Female | 0.0091 | 0.0001 |
| NHANES common sample | Survey cycle | 0.0151 | 0.0003 |
| NHANES common sample | FPG | 0.0049 | 0.0004 |
| NHANES common sample | Race or ethnicity | 0.0137 | 0.0003 |
| NHANES common sample | BMI | 0.0041 | 0.0036 |
| NHANES common sample | Waist circumference | 0.0003 | 0.0007 |
| NHANES common sample | Triglycerides | 0.0052 | 0.0009 |
| NHANES common sample | HDL-C | 0.0029 | 0.0019 |
| NHANES common sample | Systolic blood pressure | 0.0061 | 0.0008 |
| NHANES common sample | Smoking | 0.0034 | 0.0036 |
| NHANES common sample | HbA1c | 0.0024 | 0.0018 |

Selected participants are compared with the full target population, using the target SD as a fixed denominator. Categorical entries show the largest absolute SMD across levels; complete level-specific values and truncated-weight results are supplied in the aggregate CSV. This is distinct from the original available-versus-unavailable SMD of 0.047 and cannot exclude unmeasured selection.

**Supplementary Table 12b Weight diagnostics**

| **Selection mechanism** | **Target n** | **Observed n** | **Probability range** | **IPW range** | **Effective n** |
| --- | --- | --- | --- | --- | --- |
| Local HbA1c availability | 12,721 | 10,794 | 0.743 to 0.928 | 0.914 to 1.142 | 10749.4 |
| NHANES common-sample selection | 6,930 | 6,455 | 0.664 to 0.977 | 0.964 to 1.418 | 4020.4 |

Local weights apply to 12,721 adults eligible before HbA1c measurement; 10,794 have observed HbA1c, including three subsequently excluded for HbA1c-defined diabetes. The NHANES target includes 6,930 outcome-eligible adults. Effective sample size is (sum w)²/sum(w²), using survey weights multiplied by selection weights in NHANES.

**Supplementary Table 12c Local HbA1c availability by examination year**

| **Year** | **Eligible before HbA1c** | **HbA1c observed** | **HbA1c missing** | **Availability** |
| --- | --- | --- | --- | --- |
| 2020 | 1,577 | 1,198 | 379 | 76.0% |
| 2021 | 1,692 | 1,340 | 352 | 79.2% |
| 2022 | 1,800 | 1,469 | 331 | 81.6% |
| 2023 | 1,968 | 1,670 | 298 | 84.9% |
| 2024 | 1,974 | 1,735 | 239 | 87.9% |
| 2025 | 2,165 | 1,957 | 208 | 90.4% |
| 2026 | 1,545 | 1,425 | 120 | 92.2% |

The target comprises 12,721 adults meeting age, known-diabetes, pregnancy and FPG <100 mg/dL criteria before HbA1c availability is required. Of the 10,794 observed HbA1c values, three meet the HbA1c diabetes threshold and are subsequently excluded.

**Supplementary Table 13a Internal AUC sensitivity to complete-record selection**

| **Model** | **Original AUC** | **IPW at evaluation only** | **IPW at training and evaluation** |
| --- | --- | --- | --- |
| Reference | 0.7268 | 0.7276 | 0.7276 |
| + FPG | 0.7367 | 0.7373 | 0.7374 |
| + TG | 0.7270 | 0.7277 | 0.7278 |
| + FPG + TG + WHtR | 0.7460 | 0.7466 | 0.7467 |
| + TyG | 0.7280 | 0.7287 | 0.7287 |
| + TyG-BMI | 0.7399 | 0.7408 | 0.7409 |
| + TyG-WC | 0.7364 | 0.7371 | 0.7372 |
| + TyG-WHtR | 0.7387 | 0.7394 | 0.7395 |
| + METS-IR | 0.7408 | 0.7416 | 0.7417 |
| + CMI | 0.7288 | 0.7295 | 0.7295 |
| + BMI | 0.7406 | 0.7415 | 0.7416 |
| + WHtR | 0.7400 | 0.7407 | 0.7408 |

All columns use the same 6,455 participants and averaged out-of-fold predictions. Selection probabilities are estimated in training PSUs; outcomes are not used as selection-model predictors. These are descriptive sensitivity estimates without separately estimated IPW-contrast confidence intervals. Table 3 instead reports means of repeat-specific performance.

**Supplementary Table 13b Frozen external AUC sensitivity to HbA1c availability**

| **Model** | **Original AUC** | **HbA1c IPW** | **Truncated HbA1c IPW** |
| --- | --- | --- | --- |
| Reference | 0.6721 | 0.6719 | 0.6719 |
| + FPG | 0.6819 | 0.6818 | 0.6818 |
| + TG | 0.6835 | 0.6833 | 0.6833 |
| + FPG + TG + WHtR | 0.6953 | 0.6952 | 0.6952 |
| + TyG | 0.6933 | 0.6930 | 0.6930 |
| + TyG-BMI | 0.7037 | 0.7036 | 0.7036 |
| + TyG-WC | 0.7026 | 0.7024 | 0.7024 |
| + TyG-WHtR | 0.7031 | 0.7029 | 0.7029 |
| + METS-IR | 0.7101 | 0.7100 | 0.7100 |
| + CMI | 0.6906 | 0.6903 | 0.6903 |
| + BMI | 0.6921 | 0.6921 | 0.6921 |
| + WHtR | 0.6877 | 0.6876 | 0.6876 |

All columns use 8,574 participants and the same NHANES-frozen predictions. No model is refitted locally. Availability weights are truncated at the first and 99th percentiles in the final column. Other performance metrics are supplied in the aggregate CSV.

**Supplementary Table 14 Outcome-imputation and delta-adjustment sensitivity**

| **Cohort** | **Adjustment** | **HbA1c shift (pp)** | **Mean n** | **Mean outcomes** | **PR (95% CI)** | **P** |
| --- | --- | --- | --- | --- | --- | --- |
| NHANES | Model 1 | -0.5 | 6671.0 | 1152.2 | 1.298 (1.217 to 1.385) | 2e-12 |
| NHANES | Model 3 | -0.5 | 6671.0 | 1152.2 | 1.240 (1.158 to 1.327) | 1.4e-08 |
| NHANES | Model 1 | -0.4 | 6671.0 | 1152.4 | 1.298 (1.217 to 1.385) | 2e-12 |
| NHANES | Model 3 | -0.4 | 6671.0 | 1152.4 | 1.240 (1.158 to 1.328) | 1.4e-08 |
| NHANES | Model 1 | -0.3 | 6671.0 | 1152.5 | 1.298 (1.217 to 1.385) | 2e-12 |
| NHANES | Model 3 | -0.3 | 6671.0 | 1152.5 | 1.240 (1.158 to 1.328) | 1.4e-08 |
| NHANES | Model 1 | -0.2 | 6671.0 | 1152.8 | 1.298 (1.217 to 1.385) | 2e-12 |
| NHANES | Model 3 | -0.2 | 6671.0 | 1152.8 | 1.240 (1.158 to 1.327) | 1.4e-08 |
| NHANES | Model 1 | -0.1 | 6671.0 | 1153.4 | 1.298 (1.217 to 1.385) | 1.9e-12 |
| NHANES | Model 3 | -0.1 | 6671.0 | 1153.4 | 1.240 (1.158 to 1.327) | 1.4e-08 |
| NHANES | Model 1 | 0.0 | 6671.0 | 1154.2 | 1.299 (1.218 to 1.385) | 1.9e-12 |
| NHANES | Model 3 | 0.0 | 6671.0 | 1154.2 | 1.240 (1.159 to 1.328) | 1.4e-08 |
| NHANES | Model 1 | 0.1 | 6671.0 | 1155.4 | 1.298 (1.217 to 1.384) | 1.9e-12 |
| NHANES | Model 3 | 0.1 | 6671.0 | 1155.4 | 1.240 (1.158 to 1.327) | 1.4e-08 |
| NHANES | Model 1 | 0.2 | 6670.9 | 1157.1 | 1.297 (1.217 to 1.383) | 2.1e-12 |
| NHANES | Model 3 | 0.2 | 6670.9 | 1157.1 | 1.239 (1.157 to 1.326) | 1.5e-08 |
| NHANES | Model 1 | 0.3 | 6670.8 | 1159.0 | 1.296 (1.216 to 1.382) | 2.3e-12 |
| NHANES | Model 3 | 0.3 | 6670.8 | 1159.0 | 1.238 (1.156 to 1.325) | 1.7e-08 |
| NHANES | Model 1 | 0.4 | 6670.6 | 1161.3 | 1.295 (1.215 to 1.380) | 2.4e-12 |
| NHANES | Model 3 | 0.4 | 6670.6 | 1161.3 | 1.236 (1.155 to 1.323) | 1.9e-08 |
| NHANES | Model 1 | 0.5 | 6670.5 | 1163.5 | 1.293 (1.213 to 1.378) | 2.6e-12 |
| NHANES | Model 3 | 0.5 | 6670.5 | 1163.5 | 1.235 (1.154 to 1.321) | 2.1e-08 |
| Local | Model 1 | -0.5 | 11319.0 | 1042.9 | 1.481 (1.406 to 1.561) | 2.2e-48 |
| Local | Model 3 | -0.5 | 11319.0 | 1042.9 | 1.485 (1.408 to 1.565) | 7.6e-48 |
| Local | Model 1 | -0.4 | 11319.0 | 1052.8 | 1.484 (1.407 to 1.564) | 4.6e-48 |
| Local | Model 3 | -0.4 | 11319.0 | 1052.8 | 1.487 (1.410 to 1.568) | 1.2e-47 |
| Local | Model 1 | -0.3 | 11318.9 | 1071.4 | 1.489 (1.413 to 1.569) | 3.4e-49 |
| Local | Model 3 | -0.3 | 11318.9 | 1071.4 | 1.492 (1.415 to 1.573) | 2e-48 |
| Local | Model 1 | -0.2 | 11318.9 | 1101.3 | 1.487 (1.411 to 1.567) | 4.2e-49 |
| Local | Model 3 | -0.2 | 11318.9 | 1101.3 | 1.490 (1.413 to 1.571) | 3.9e-48 |
| Local | Model 1 | -0.1 | 11318.9 | 1149.2 | 1.487 (1.411 to 1.567) | 5.2e-48 |
| Local | Model 3 | -0.1 | 11318.9 | 1149.2 | 1.489 (1.412 to 1.571) | 1.3e-46 |
| Local | Model 1 | 0.0 | 11318.6 | 1219.6 | 1.483 (1.408 to 1.562) | 6.9e-47 |
| Local | Model 3 | 0.0 | 11318.6 | 1219.6 | 1.485 (1.409 to 1.565) | 5.3e-46 |
| Local | Model 1 | 0.1 | 11318.0 | 1323.9 | 1.469 (1.396 to 1.547) | 1.4e-43 |
| Local | Model 3 | 0.1 | 11318.0 | 1323.9 | 1.471 (1.396 to 1.550) | 1.2e-42 |
| Local | Model 1 | 0.2 | 11316.0 | 1458.4 | 1.442 (1.371 to 1.517) | 7.3e-40 |
| Local | Model 3 | 0.2 | 11316.0 | 1458.4 | 1.443 (1.371 to 1.519) | 9.2e-39 |
| Local | Model 1 | 0.3 | 11312.1 | 1606.6 | 1.411 (1.345 to 1.481) | 1.9e-38 |
| Local | Model 3 | 0.3 | 11312.1 | 1606.6 | 1.411 (1.343 to 1.483) | 1.1e-36 |
| Local | Model 1 | 0.4 | 11302.2 | 1769.8 | 1.371 (1.310 to 1.435) | 7.1e-37 |
| Local | Model 3 | 0.4 | 11302.2 | 1769.8 | 1.371 (1.308 to 1.436) | 7.4e-35 |
| Local | Model 1 | 0.5 | 11283.5 | 1947.9 | 1.330 (1.275 to 1.387) | 1.6e-36 |
| Local | Model 3 | 0.5 | 11283.5 | 1947.9 | 1.329 (1.273 to 1.388) | 1.2e-34 |

Model 1 adjusts for age, sex and NHANES cycle or local enrolment year. Model 2 additionally adjusts for education, income-to-poverty ratio in NHANES or income category locally, smoking, alcohol use, and vigorous activity in NHANES or physical activity locally. Model 3 additionally adjusts for systolic blood pressure, hypertension, lipid-lowering medication and eGFR, with family history of diabetes included locally. PRs are per cohort-specific standard-deviation increase. NHANES uses design-based standard errors; local estimates use HC0 sandwich standard errors. Fifty imputations and 20 iterations were used. Missing HbA1c, but no index components, was imputed. The delta shift is applied only to missing HbA1c; delta zero corresponds to MAR. HbA1c-defined diabetes is excluded after reclassification. Decimal rounding prevents floating-point artefacts at diagnostic thresholds. The NHANES pool has 6,679 adults with 20 missing HbA1c values; the local pool has 11,322 adults with 1,705 missing values. Fractional n and outcome counts are averages across imputations. Scenarios do not establish that missingness is MAR or that other MNAR mechanisms are harmless.

**Supplementary Table 15 HbA1c-defined prediabetes by cohort-wide TyG-WHtR quartile**

| **Cohort** | **Group** | **Quartile** | **n** | **Outcomes** | **Prevalence (95% CI)** |
| --- | --- | --- | --- | --- | --- |
| NHANES | All adults | Q1 | 1,496 | 134 | 6.1% (4.9 to 7.7) |
| NHANES | All adults | Q2 | 1,653 | 246 | 11.2% (9.4 to 13.3) |
| NHANES | All adults | Q3 | 1,713 | 336 | 15.2% (13.3 to 17.3) |
| NHANES | All adults | Q4 | 1,789 | 436 | 18.3% (16.5 to 20.2) |
| NHANES | Women | Q1 | 865 | 54 | 4.4% (3.1 to 6.3) |
| NHANES | Women | Q2 | 950 | 127 | 10.8% (8.4 to 13.8) |
| NHANES | Women | Q3 | 961 | 198 | 17.4% (14.5 to 20.6) |
| NHANES | Women | Q4 | 1,131 | 304 | 21.3% (18.8 to 24.0) |
| NHANES | Men | Q1 | 631 | 80 | 8.7% (6.5 to 11.6) |
| NHANES | Men | Q2 | 703 | 119 | 11.8% (9.2 to 14.9) |
| NHANES | Men | Q3 | 752 | 138 | 12.4% (10.0 to 15.3) |
| NHANES | Men | Q4 | 658 | 132 | 13.6% (10.9 to 16.8) |
| NHANES | <50 years | Q1 | 1,227 | 75 | 4.3% (3.3 to 5.7) |
| NHANES | <50 years | Q2 | 1,109 | 89 | 6.0% (4.6 to 7.7) |
| NHANES | <50 years | Q3 | 1,005 | 119 | 9.2% (7.5 to 11.2) |
| NHANES | <50 years | Q4 | 1,054 | 169 | 12.4% (10.5 to 14.5) |
| NHANES | >=50 years | Q1 | 269 | 59 | 14.1% (10.0 to 19.7) |
| NHANES | >=50 years | Q2 | 544 | 157 | 23.4% (18.6 to 29.0) |
| NHANES | >=50 years | Q3 | 708 | 217 | 25.5% (21.5 to 30.0) |
| NHANES | >=50 years | Q4 | 735 | 267 | 28.3% (24.4 to 32.6) |
| Local | All adults | Q1 | 2,404 | 140 | 5.8% (5.0 to 6.8) |
| Local | All adults | Q2 | 2,403 | 224 | 9.3% (8.2 to 10.5) |
| Local | All adults | Q3 | 2,403 | 257 | 10.7% (9.5 to 12.0) |
| Local | All adults | Q4 | 2,404 | 415 | 17.3% (15.8 to 18.8) |
| Local | Men | Q1 | 1,076 | 54 | 5.0% (3.9 to 6.5) |
| Local | Men | Q2 | 1,141 | 101 | 8.9% (7.3 to 10.6) |
| Local | Men | Q3 | 1,199 | 129 | 10.8% (9.1 to 12.6) |
| Local | Men | Q4 | 1,255 | 212 | 16.9% (14.9 to 19.1) |
| Local | Women | Q1 | 1,328 | 86 | 6.5% (5.3 to 7.9) |
| Local | Women | Q2 | 1,262 | 123 | 9.7% (8.2 to 11.5) |
| Local | Women | Q3 | 1,204 | 128 | 10.6% (9.0 to 12.5) |
| Local | Women | Q4 | 1,149 | 203 | 17.7% (15.6 to 20.0) |
| Local | <50 years | Q1 | 1,204 | 29 | 2.4% (1.7 to 3.4) |
| Local | <50 years | Q2 | 1,171 | 57 | 4.9% (3.8 to 6.3) |
| Local | <50 years | Q3 | 1,242 | 72 | 5.8% (4.6 to 7.2) |
| Local | <50 years | Q4 | 1,177 | 123 | 10.5% (8.8 to 12.3) |
| Local | >=50 years | Q1 | 1,200 | 111 | 9.2% (7.7 to 11.0) |
| Local | >=50 years | Q2 | 1,232 | 167 | 13.6% (11.8 to 15.6) |
| Local | >=50 years | Q3 | 1,161 | 185 | 15.9% (13.9 to 18.2) |
| Local | >=50 years | Q4 | 1,227 | 292 | 23.8% (21.5 to 26.3) |

Quartile boundaries are calculated once per cohort, with survey weights in NHANES, and are not recalculated by sex or age. NHANES prevalence and logit confidence intervals use the survey design; local intervals are Wilson intervals. Counts are unweighted. These unadjusted summaries are descriptive and do not constitute interaction tests.

**Supplementary Table 16 All candidate models including BMI and WHtR additions**

| **Model** | **Internal mean-repeat AUC** | **External AUC (95% CI)** | **Status** |
| --- | --- | --- | --- |
| Reference | 0.727 | 0.672 (0.655 to 0.690) | Original |
| + FPG | 0.737 | 0.682 (0.665 to 0.699) | Original |
| + TG | 0.727 | 0.684 (0.666 to 0.701) | Original |
| + FPG + TG + WHtR | 0.746 | 0.695 (0.678 to 0.712) | Original |
| + TyG | 0.728 | 0.693 (0.676 to 0.710) | Original |
| + TyG-BMI | 0.740 | 0.704 (0.687 to 0.721) | Original |
| + TyG-WC | 0.736 | 0.703 (0.686 to 0.719) | Original |
| + TyG-WHtR | 0.739 | 0.703 (0.686 to 0.720) | Original |
| + METS-IR | 0.741 | 0.710 (0.693 to 0.727) | Original |
| + CMI | 0.729 | 0.691 (0.673 to 0.708) | Original |
| + BMI | 0.740 | 0.692 (0.675 to 0.709) | Added secondary |
| + WHtR | 0.740 | 0.688 (0.670 to 0.705) | Added secondary |

Every candidate uses the original common samples and folds. New BMI and WHtR models are fitted and standardised only in NHANES, then applied without local fitting. Their inclusion is secondary and does not change the original frozen models.

**Supplementary Table 17 Retrospective testing trade-offs at frozen thresholds**

| **Model** | **Threshold** | **Selected for HbA1c** | **Detected / 928 outcomes** | **Missed / 928 outcomes** | **Tests per 1,000** | **Missed outcomes per 1,000** |
| --- | --- | --- | --- | --- | --- | --- |
| Reference | 0.1334 | 4,858 | 722 | 206 | 566.6 | 24.0 |
| + FPG | 0.1200 | 3,449 | 588 | 340 | 402.3 | 39.7 |
| + TG | 0.1288 | 5,120 | 752 | 176 | 597.2 | 20.5 |
| + FPG + TG + WHtR | 0.1192 | 2,796 | 515 | 413 | 326.1 | 48.2 |
| + TyG | 0.1312 | 5,026 | 757 | 171 | 586.2 | 19.9 |
| + TyG-BMI | 0.1160 | 4,792 | 747 | 181 | 558.9 | 21.1 |
| + TyG-WC | 0.1160 | 4,338 | 710 | 218 | 505.9 | 25.4 |
| + TyG-WHtR | 0.1205 | 4,265 | 704 | 224 | 497.4 | 26.1 |
| + METS-IR | 0.1234 | 4,565 | 741 | 187 | 532.4 | 21.8 |
| + CMI | 0.1301 | 5,017 | 755 | 173 | 585.1 | 20.2 |

Denominator is 8,574 adults. All thresholds are the original NHANES-derived model probability thresholds, used at full numerical precision. Universal HbA1c testing would test all 8,574 and ascertain all 928 observed study outcomes by definition. Counts describe retrospective classification, not an implemented testing programme, observed test reduction, cost-effectiveness or improved clinical outcomes. Inputs are assumed to be available; their costs are not evaluated.

# Supplementary figures

**Supplementary Fig. 1 | Decision curves in repeated internal validation.** Net benefit is shown for ordering HbA1c across thresholds from 5% to 25%. Curves use averaged out-of-fold NHANES predictions and are exploratory.

**Supplementary Fig. 2 | Subgroup associations of TyG-WHtR.** Points and bars show prevalence ratios and 95% CIs per cohort-specific standard deviation. Models adjust for age, sex and survey cycle or enrolment year, with age omitted for age strata and sex omitted for sex strata. Sample and outcome counts are listed in Supplementary Table 4. Interaction P values use Benjamini–Hochberg false-discovery-rate adjustment within the original cohort-specific families. Findings are hypothesis-generating.

**Supplementary Fig. 3 | Multiple-imputation diagnostics.** The six panels show traces for selected covariates in the primary covariate-imputation analysis. Lines are means across 50 chains; bands are the 10th–90th percentiles of chain means, not confidence intervals. There were 20 iterations. HbA1c outcomes were not imputed in this primary analysis.

**Supplementary Fig. 4 | Convergence with HOMA-IR.** Correlations compare TyG-WHtR and other indices with log-transformed HOMA-IR where fasting insulin was available. Shared metabolic inputs preclude mechanistic interpretation.

Supplementary Figure 5 | Measured balance before and after selection weighting. a, NHANES common prediction sample (n=6,455) relative to the outcome-eligible target (n=6,930). b, Local adults with observed HbA1c (n=10,794) relative to the pre-HbA1c eligibility population (n=12,721). Points show absolute standardised differences before and after IPW, using a fixed target-population SD. For categorical variables, the maximum across levels is displayed. NHANES summaries include survey weights. These are descriptive balance diagnostics without hypothesis tests; small measured differences do not exclude unmeasured selection. Detailed definitions and weight diagnostics are given in Supplementary Table 12.

Supplementary Figure 6 | Outcome prevalence across cohort-wide TyG-WHtR quartiles by sex and age. a and b, Sex-stratified NHANES and local prevalence. c and d, Age-stratified NHANES and local prevalence. Quartiles use the overall cohort distribution and are not recalculated within subgroups. The index-complete samples include 6,651 NHANES participants with 1,152 outcomes and 9,614 local participants with 1,036 outcomes. Points indicate unadjusted prevalence; error bars indicate 95% CIs. NHANES estimates and logit intervals account for the fasting survey design; local intervals use the Wilson method. Lines connect descriptive estimates and do not represent fitted trends. Exact quartile and subgroup counts appear in Supplementary Table 15. These plots are not interaction tests; the adjusted subgroup signals remain hypothesis-generating.

Supplementary Figure 7 | Population composition across TyG-WHtR quartiles. a, NHANES 2005–2018. b, Local cohort. Each series shows the female share or the share aged under 50 years within each cohort-wide quartile. These two characteristics overlap and the series are not intended to sum to 100%. NHANES proportions are survey weighted; local proportions are unweighted. Counts and quartile denominators are provided in the accompanying source data. The samples and quartile definitions match Supplementary Figure 6. These are descriptive population summaries without inferential intervals or subgroup-effect tests.

Supplementary Figure 8 | Fully adjusted associations under outcome-imputation assumptions. a, NHANES 2005–2018, with 20 missing HbA1c values in an initial pool of 6,679 adults. b, Local cohort, with 1,705 missing HbA1c values in an initial pool of 11,322 adults. Points show Model 3 PRs and error bars show Rubin-pooled 95% CIs from 50 imputations and 20 iterations. Delta zero corresponds to the MAR analysis. The horizontal axis is an assumed additive shift in HbA1c percentage points applied only to missing values; index components are not imputed. Outcomes are reclassified and HbA1c-defined diabetes is excluded after each shift. The horizontal dashed line indicates PR=1. Covariates and varying analytic counts are specified in Supplementary Table 14. The shift grid is a stress test and does not validate MAR, estimate the true missingness mechanism or cover every MNAR mechanism.
